# Diverse Roles of MAX1 Homologues in Rice

**DOI:** 10.3390/genes11111348

**Published:** 2020-11-13

**Authors:** Marek Marzec, Apriadi Situmorang, Philip B. Brewer, Agnieszka Brąszewska

**Affiliations:** 1 Faculty of Natural Sciences, Institute of Biology, Biotechnology and Environmental Protection, University of Silesia in Katowice, Jagiellonska 28, 40-032 Katowice, Poland; agnieszka.braszewska-zalewska@us.edu.pl; 2 ARC Centre of Excellence in Plant Energy Biology, Waite Research Institute, School of Agriculture, Food and Wine, The University of Adelaide, Glen Osmond, SA 5064, Australia; apriadi.situmorang@adelaide.edu.au (A.S.); philip.brewer@adelaide.edu.au (P.B.B.)

**Keywords:** co-expression gene network, developmental processes, gene expression in silico, microRNA, stress response, strigolactones, transcription factors

## Abstract

Cytochrome P450 enzymes encoded by *MORE AXILLARY GROWTH1* (*MAX1*)-like genes produce most of the structural diversity of strigolactones during the final steps of strigolactone biosynthesis. The diverse copies of *MAX1* in *Oryza sativa* provide a resource to investigate why plants produce such a wide range of strigolactones. Here we performed in silico analyses of transcription factors and microRNAs that may regulate each rice *MAX1*, and compared the results with available data about *MAX1* expression profiles and genes co-expressed with *MAX1* genes. Data suggest that distinct mechanisms regulate the expression of each *MAX1*. Moreover, there may be novel functions for *MAX1* homologues, such as the regulation of flower development or responses to heavy metals. In addition, individual *MAX1s* could be involved in specific functions, such as the regulation of seed development or wax synthesis in rice. Our analysis reveals potential new avenues of strigolactone research that may otherwise not be obvious.

## 1. Introduction

Strigolactones (SLs) were first discovered as stimulators of seed germination in species of the genera *Orobanche*, *Phelipanche* and *Striga* [1]. Then, exudation of SLs from roots was found to promote hyphal branching of arbuscular mycorrhizal (AM) fungi [2]. More recently, SLs were described as a novel group of endogenous plant hormones, based on the analysis of mutants with semi-dwarf and high-branched phenotype [3,4]. Henceforth, additional roles of SLs in plant growth and development, such as the regulation of the root system development [5], elongation of the mesocotyl and stem [6,7], vasculature formation and secondary growth [8,9] and leaf senescence [10], were discovered. Finally, it was postulated that SLs are involved in plant responses to various biotic [11,12] and abiotic [13] stresses. Most SL biosynthesis appears to occur in vasculature, with resultant transport upwards in shoots or exudation out of roots [14].

**Figure 1:**
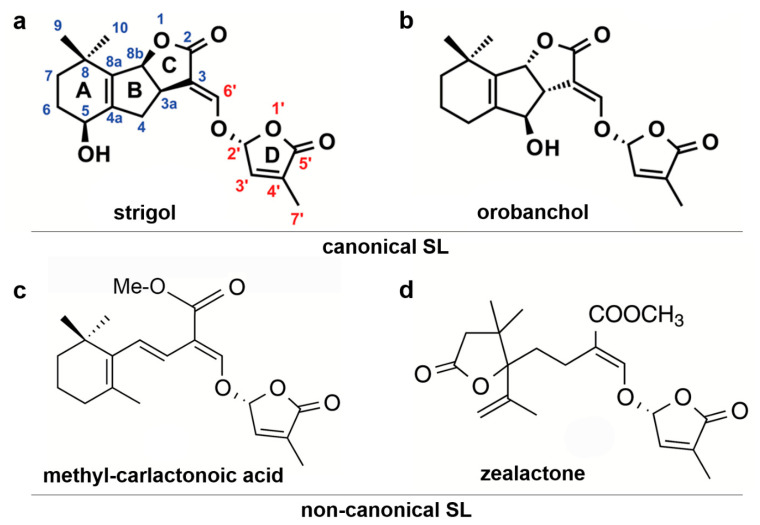
Structures of strigolactones (SLs). (**a**,**b**) Canonical SLs and (**c**,**d**) non-canonical SLs.

More than 30 natural SLs have been identified so far, and are classified into two groups, canonical SLs and non-canonical SLs, based on their chemical structures [15] (Figure 1). All 23 so-far-identified canonical SLs contain ABC-rings connected, via an enol-ether bridge, to a methylbutenolide D-ring [16]. Conversely, a growing list of non-canonical SLs do not contain the ABCD-ring structure (Figure 1c,d) [15,17]. Non-canonical SLs were revealed for the first time when carlactone (CL) was identified [18], which was later found to be a precursor for methyl-carlactonoic acid [19] (Figure 1c). Next, zealactone [20,21] (Figure 1d) and avenaol [22] were isolated from root exudates of maize (*Zea mays*) and black oat (*Avena strigose*), respectively. These non-canonical SL lack A-, B- and C-ring but still exhibited activity during parasitic weed seed germination stimulation, which is dependent on the presence of the enol-ether-D-ring moiety in SL compounds [20,21]. This is why it is now postulated that SLs should be defined as bioactive ‘carotenoid-derived molecules with a butenolide D-ring’ [23].

Canonical SL can be divided into two subgroups, strigol-type and orobanchol-type, based on the stereochemistry of the C-ring (Figure 1a,b). Strigol-type SLs have a β-oriented C-ring (3a*R*,8b*S*), and orobanchol-type SLs have an α-oriented C-ring (3a*S*,8b*R*) [23]. So far, in all examined exudates of different plants, a mixture of more than one SLs was identified [summarised by [23]. Various SL profiles were described for different species, different varieties of the same species and even for the same variety exposed to different growing conditions or at different developmental stages [24,25]. Some plant species, such as rice (*Oryza sativa*), do not produce strigol-type SLs, whereas the other plant species such as tobacco (*Nicotiana tabacum*) produce both, orobanchol- and strigol-type SLs [26]. Also, plants that produce both canonical and non-canonical SL were described (reviewed by [23]).

Structural diversity of SLs raises questions about the effect of the chemical structure on biological activities of SL compounds. For all the described SLs, a stimulus effect on parasitic weed seed germination, induction of hyphal branching of AM fungi and inhibition of plant shoot branching have been shown. However, different SLs exhibited various efficiency in these processes. For example, in garden pea (*Pisum sativum*), strigol and orobanchol showed less activity in inhibition of shoot branching in comparison to orobanchyl acetate and 5-deoxystrigol [27].

**Figure 2:**
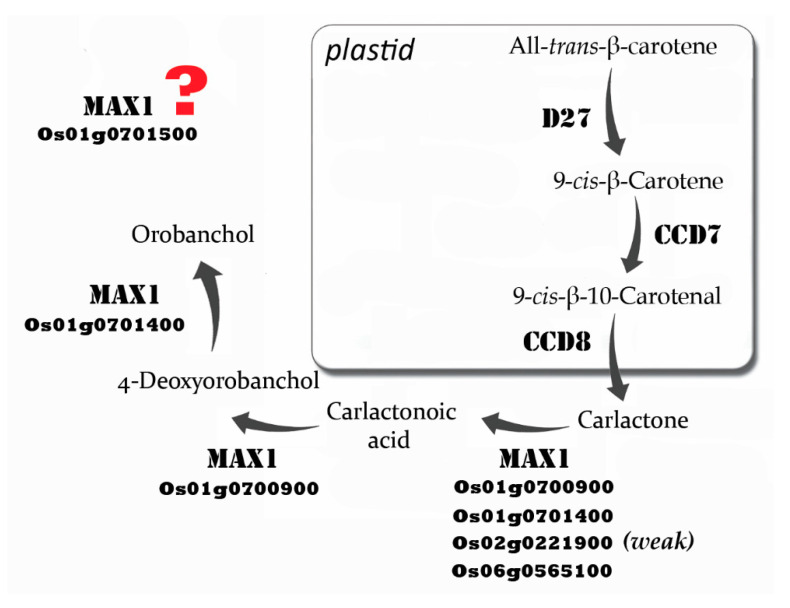
SL biosynthesis pathway in rice.

The large number of SLs that are produced in plants lies in contrast to the low number of components for SL biosynthesis (reviewed by [28]) and signalling (reviewed by [29,30]) pathways. The first steps of SL biosynthesis occur in plastid membranes where the D27 (DWARF27) carotenoid isomerase converts all-*trans*-β-carotene into 9-*cis*-β-carotene (Figure 2). 9-*cis*-β-Carotene is a substrate for the next stages of SL production conducted by carotenoid cleavage dioxygenases (CCDs). The first one, CCD7, is a stereo-specific dioxygenase that cleaves 9-*cis*-β-carotene to produce 9-*cis*-β-10-carotenal, which is subsequently processed by CCD8 to produce CL [18]. Genes that encode D27 and both CCDs have been identified in many different plant species, including moss (*Physcomitrella patens*). However, a different number of gene copies encoding these proteins was identified in different species. For example, two, four and six copies of *CCD8* were found in maize, rice and sorghum (*Sorghum bicolor*), respectively (reviewed by [23]). The plastidic pathway of SL biosynthesis ends with CL production and, based on grafting studies, CL and downstream products are free to move out of cells [31,32,33]. The structure of CL is similar to that described for canonical and non-canonical SLs, because it consists of a C19-skeleton and a C14-moiety that corresponds to the D-ring of SLs [18].

The next stages of SL biosynthesis are different in the two model species, *Arabidopsis thaliana* and rice, yet many of the steps are provided by MAX1 (MORE AXILLARY GROWTH1)-like enzymes that are members of the CYP711A cytochrome P450 family. In *A. thaliana* only one copy of *MAX1* was identified and the enzyme converts CL into carlactonoic acid (9-desmethyl-9-carboxy-carlactone; CLA) [19,31]. CLA is then converted by un unknown methyl transferase to methyl carlactonoate (MeCLA) [19], which is next processed into hydroxyl-methyl-carlactonoate (1′-HO-MeCLA) by a 2-oxoglutarate-dependent dioxygenase, LATERAL BRANCHING OXIDOREDUCTASE (LBO) [17,33].

The CL to CLA reaction seems to occur in all plant species so far tested [34] and CLA now appears to be the universal precursor for all SLs—both canonical and non-canonical [34]. CLA is probably not bioactive, but is converted into bioactive SLs [19]. The production of canonical SLs seems to be absent in *A. thaliana* [35]. The *max1* knockout mutant phenotypes in *A. thaliana*, rapeseed (double mutant) and tomato appear strong, suggesting that most, if not all, SL biosynthesis occurs via MAX1 [31,36,37]. CLA variants hydroylated at the A-ring have also been detected [17]. It is unclear if these CLA variants come from carotenoid precursors via the CCDs or if they are produced from CLA by other enzymes. MAX1 from *Lotus japonicus* actually converts CLA to a CLA variant hydroylated at the A-ring, at C-18 [38]. This 18-hydroxy-CLA is further converted to 5-deoxystrigol and lotuslactone by unknown enzymes. A second *L. japonicus* MAX1 has not yet been tested [38], but is a good candidate for further biosynthesis.

**Figure 3:**
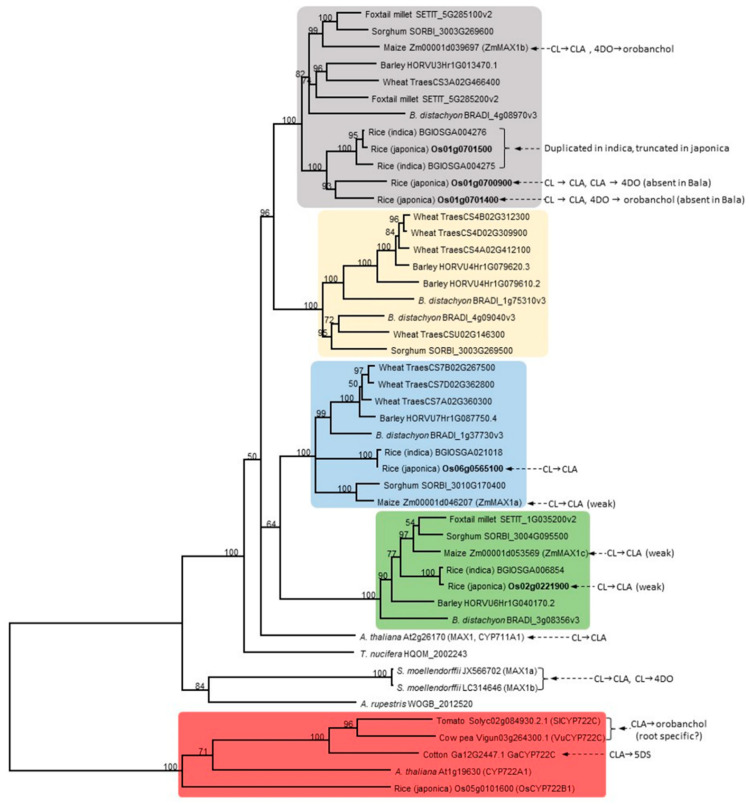
Phylogenetic relationship of MAX1 amino acid sequences from rice (*Oryza sativa*, subsp. *japonica* and *indica*), barley (*Hordeum vulgare*), wheat (*Triticum aestivum*), sorghum (*Sorghum bicolor*), maize (*Zea mays*), foxtail millet (*Setaria italica*), *Brachypodium distachyon*, *A. thaliana*, conifer *Torreya nucifera*, fern ally *Selaginalla moellendorffii* and moss *Andreaea rupestris*. The tree is rooted with selected CYP722C members (red) and annotated with known enzyme reactions (CL = carlactone, CLA = carlactonoic acid, 4DO = 4-deoxyorobanchol, 5DS = 5-deoxystrigol). Bootstrap percentage values shown and highlighted groups indicate distinctive clades.

Five copies of *MAX1* in rice were identified, *Os01g0700900*, *Os01g0701400*, *Os01g0701500*, *Os02g0221900* and *Os06g0565100* (Figure 2). CL was converted into CLA by Os01g0700900, Os01g0701400, Os02g0221900 or Os06g0565100 (live yeast cell assays), but the activity of Os02g0221900 was very weak and Os01g0701500 was absent [35]. Interestingly, a maize protein closely related to Os02g0221900 also showed only very weak CL to CLA activity [35]. This Os02g0221900 sub-clade is quite distinct (Figure 3), so it will be important to discover if the members have any other distinctive function. Note that cytochrome P450s enzymatic assays require microsomes or live cells (yeast, *E. coli* or insect) (MAX1 enzymes have an N-terminal transmembrane domain) and co-expression/incubation with an NADPH-P450 reductase.

Remarkably, in addition to making CLA, Os01g0700900 also independently closes the B-C rings of CLA to make 4-deoxyorobanchol (4DO; also called *ent*-2′-*epi*-5-deoxystrigol) [19,35]. Os01g0701400 can independently convert 4DO to orobanchol via hydroxylation [19,35,39]. It is unknown whether Os01g0701500, Os02g0221900 or Os06g0565100 perform steps other than CL to CLA or whether Os01g0701500 is functional (see below). The Bala indica rice cultivar (and most indica varieties) has a deletion of *Os01g0700900* and *Os01g0701400* (and duplication of *Os01g0701500*), is high tillering, semi-dwarf and defective in SL production, yet it still produces a small amount of SLs, suggesting partial redundancy by other enzymes [40].

Non-MAX1 cytochrome P450s may also act in SL biosynthesis, and may fill this redundancy role. CYP722C from cowpea and tomato can convert CLA into orobanchol, probably via 18-hydroxy-CLA [41] (Figure 3). The knockout mutant in tomato had normal branching and normal SL feedback, but root exudates were deficient in orobanchol and solanacol, and were poor in germinating parasitic weed seeds [41]. Roots showed an increase in CLA [41], suggesting a specific function for converting CLA to orobanchol in roots. In contrast, CYP722C from cotton converts CLA into 5-deoxystrigol via 18-hydroxy-CLA [42]. Other families of enzymes, such as 2-oxoglutarate-dependent dioxygenases, that include LBO [33], have potential to act in combination with MAX1s in SL biosynthesis [36].

Complementation studies with the *A. thaliana max1-1* mutant using *MAX1* rice sequences revealed that *Os01g0700900* and *Os01g0701400* [40] and *Os02g0221900* and *Os06g0565100* [43], were able to rescue *max1-1*. Whereas overexpression of *Os01g0701500* in the *max1-1* background did not rescue the mutant phenotype, which might be explained by the presence of the premature stop codon 20 residues from the end of sequences [43]. Three other SLs with unknown structure were detected in rice exudates and tentatively named methoxy-5-deoxystrigol (Me-O-5-DS) isomers [40]. However, these are likely non-canonical SLs with currently unknown biosynthesis. In addition, it is still unknown whether CLA is converted to MeCLA in rice. Added CLA was converted to MeCLA in sunflower [34], but endogenous MeCLA has otherwise only been detected in *A. thaliana* and poplar [23], and the methyl transferase that makes MeCLA from CLA remains unknown. MeCLA has been proposed to be a precursor for zealactone and was recently confirmed as the precursor for heliolactone [23,44]. It will be important to discover if MeCLA exists in rice and if it is a substrate for rice LBO.

Strigolactone variants may have evolved to not attract parasitic weeds but still attract beneficial soil microbes. Different strigolactones may also vary in their specificity for plant traits, such as branching [27]. These complex selective pressures may result in diversification of cytochrome P450s. Mutations in new gene copies may alter enzyme function or expression.

Our knowledge of the enzymes involved in the structural diversification of SLs remains scarce. Because different SLs have different biological activities, it will be crucial to identify the components involved in the synthesis of each SL and find out if they have tissue-specific regulation. This will allow us to investigate the role of SLs in plants with much higher precision. With that in mind, we performed in silico analyses using all five rice *MAX1* homologues to uncover regulatory mechanisms that may control their expression. Together with the analysis of their expression patterns during plant development and responses to various factors, it is possible to predict further roles of rice *MAX1* homologues. These results might help us better understand the structural diversification of SLs and better elucidate the regulation of each enzymatic step.

## 2. Materials and Methods

### 2.1. Rice MAX1 Sequences and Conserved Domain Identification

Genomic, coding and protein sequences of rice *MAX1* homologues were obtained from NCBI database (www.ncbi.nlm.nih.gov): *Os01g0700900*—gene ID 4326926; *Os01g0701400*—gene ID 9269315; *Os01g0701500*—gene ID 4326929; *Os02g0221900*—gene ID 4328761; *Os06g0565100*—gene ID 4341325.

Sequence of the promotor region of rice genes were obtained using PlantPAN 3.0 platform (http://plantpan.itps.ncku.edu.tw). For each of gene the sequence of 2000 nucleotide (nt) upstream transcription start site and 500 nt downstream transcription start site were obtained.

The amino acid sequence of AtMAX1 was used as a query for BLAST search on Ensembl Plants (https://plants.ensembl.org/Multi/Tools/Blast) to gather sequences for the phylogenetic tree (Figure 3). Genes with less than 400 amino acids were excluded. MAX1 homologues from maize and *Selaginella moellendorffii* are the same as previously reported [35]. Amino acid sequences of MAX1 homologs of *Andreaea rupestris* and *Torreya nucifera* were obtained from 1KP projects https://db.cngb.org/onekp/ [45]. Amino acid sequences for CYP772 clade are the same as previously reported [41,42]. MAFFT was used for multiple sequence alignment [46]. The phylogenetic tree was constructed using Geneious Tree Builder (Geneious 8.1.9) following the Neighbour-joining method with bootstrap resampling of 1000 replicates and rooted to CYP772 clade. Sequences, alignment and gene IDs are available in Supplementary Data (Supplementary File 1).

### 2.2. Searching of Transcription Factor Motifs in Promoter Region of Rice MAX1 Genes

To predict the sites recognised by transcription factors (TFs), 2500 nt of promoter region of all five rice *MAX1* genes was used as a query in tool ‘Promoter Analysis’ implemented in PlantPAN 3.0 platform (http://plantpan.itps.ncku.edu.tw). In each case, only the database for rice-specific TFs was selected and no user-customised motifs were used. The function of TFs that are specific only to a single *MAX1* homologue, was identified based on available literature and TF databases, such as: PlantTFDB (http://planttfdb.cbi.pku.edu.cn), CIS-BP Database (http://cisbp.ccbr.utoronto.ca), New PLACE (https://www.dna.affrc.go.jp/PLACE/) and JASPAR (http://jaspar.genereg.net).

### 2.3. Identification of miRNA that Regulate MAX1 Genes

For identification of miRNA that may regulate the *MAX1* genes, the psRNATarget web server was used (http://plantgrn.noble.org/psRNATarget, 2017 release) [47]. The coding sequence of each *MAX1* gene was used as a query and only rice-specific miRNA was searched for, according to these rules: penalty for G:U pair: 0.5; penalty for other mismatches: 1; extra weight in seed region: 1.5; seed region: 2–13 nt; # of mismatches allowed in seed region: 2; HSP size: 19; penalty for opening gap: 2; penalty for extending gap: 0.5. Only results with a final score (expectation value) up to 5 were considered for further investigation. Expectation value is the penalty for the mismatches between mature small RNA and the target sequence. A higher value indicates less similarity (and possibility) between small RNA and the target candidate. The function of miRNAs that are specific only to a single *MAX1* homologue was identified based on the available literature.

### 2.4. Profile Expression of MAX1 Genes

Data of *MAX1* genes expression were obtained from the Rice Expression Database (http://expression.ic4r.org) [48] that integrates expression profiles derived entirely from NGS RNA-Seq data of rice (Nipponbare variety). The following Loc_IDs: LOC_Os01g50520, LOC_Os01g50580, LOC_Os01g50590, LOC_Os02g12890 and LOC_Os06g36920 were used for *Os01g0700900*, *Os01g0701400*, *Os01g0701500*, *Os02g0221900*, *Os06g0565100*, respectively. Only experiments containing data for all of the five *MAX1* homologues were used for *MAX1* profile expression comparisons.

### 2.5. Gene Co-Expression Network of Rice MAX1 Homologues

Lists of genes co-expressed with *MAX1* homologues were obtained using RiceFREND (https://ricefrend.dna.affrc.go.jp) [49]. The locus ID of each gene was used for the ‘single guide gene’ searching option. Only genes with a Mutual Rank (MR) lower than 100 were taken for further investigation. MR is calculated as the geometric mean of the correlation rank of gene A to gene B and of gene B to gene A, based on 24 datasets representing 815 microarray data including redundant data among the datasets. Details on each dataset can be accessed from the RiceXPro database (https://ricexpro.dna.affrc.go.jp). The data have also been deposited in NCBI’s Gene Expression Omnibus (GEO) and are accessible through GEO series accession numbers, GSE21396, GSE21397, GSE36040, GSE36042, GSE36043, GSE36044, GSE39423, GSE39424, GSE39425, GSE39426, GSE39427, GSE39429 and GSE39432. Function of selected genes was described based on available literature.

### 2.6. Gene Ontology of Genes Co-Expressed with Rice MAX1 Homologues

Gene Ontology (GO) for all genes with MR < 100 from each CGN of *MAX1s* was performed using AgriGO v2 database (http://systemsbiology.cau.edu.cn/agriGOv2) [50]. Dataset of *Oryza sativa japonica*, was screened using standard parameters: Fisher statistical test, significance level—0.05; multi adjustment method: Yekutieli (FDR under dependency).

## 3. Results

Protein sequences of rice MAX1 homologues exhibited a range of identity from 48 to 80.8%, and in all sequences, the conserved domain for cytochrome P450 (pfam00067) was present (Figure S1). We collected MAX1s amino acid sequences from plant species and produced a new phylogenetic tree with a focus on grasses (Figure 3). In terms of the MAX1 copies that we see in rice and other well-known grass species, there appears to be diversification into four distinct MAX1 clades [35,45] (Figure 3). The role of the more-distantly related CYP722Cs in grasses is unknown, but they may constitute a fifth clade (Figure 3). Have these clades evolved distinct and conserved enzymatic functions in grasses? There is some information from enzymatic studies in heterologous expression systems, summarised in Figure 3. Many MAX1s can catalyse CL to CLA. Some only do this weakly, such as those from the ‘blue’ group. The ‘grey’ group seems to have an emphasis on extra specific steps for making orobanchol-type SLs. However, at this stage, there are so many missing enzymatic steps for specific SLs from each grass species that it is difficult to generalise. It could equally be that each MAX1 has evolved its own function independently such that ancestral functions are not obvious or lost. Then there is the added complication about where each SL biosynthesis enzyme is expressed. For example, it will be interesting to investigate whether CYP722C [41] homologues are active in rice and have tissue-specific expression and function. Previously it was reported that Os01g0701500 has no enzymatic activity, because this sequence was unable to rescue phenotype of *max1-1* mutant in *A. thaliana* [43] and this protein did not exhibit enzymatic activities described for other MAX1s [35]. It was postulated that a lack of enzymatic activity of Os01g0701500 is due to premature stop codon, in comparison to the other MAX1 protein (Figure S1). On the other hand still, the full sequence of the conserved domain for cytochrome P450 is present in Os01g0701500 (Figure S1). This is why Os01g0701500 was included in presented studies.

### 3.1. Transcription Factor Motifs That Are Present in the Promoter Region of Rice MAX1 Genes

**Figure 4:**
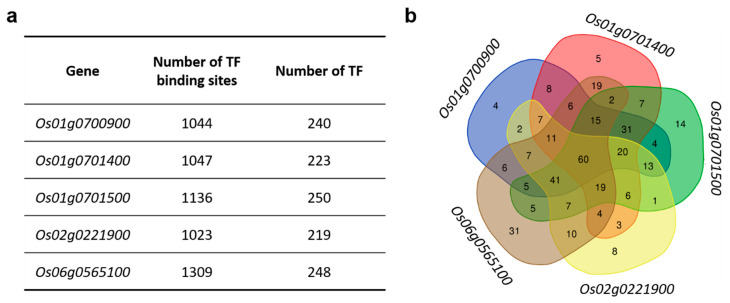
Motifs recognised by transcription factors (TFs) that are present in the promoter region of rice *MAX1* genes. (**a**) Number of TF binding sites and TFs that bind to motifs identified in promoter region of analysed genes. (**b**) The Venn diagram illustrates TFs that are specific to each of rice *MAX1* genes and those that are common for different *MAX1* homologues.

The promoter region of all five rice *MAX1* genes (*Os01g0700900*, *Os01g0701400*, *Os01g0701500*, *Os02g0221900*, *Os06g0565100*) were screened to identify sequences recognised by transcription factors (TFs). In each case, sequence of 2000 nucleotide (nt) upstream transcription start site and 500 nt downstream transcription start site was used. Various number of motifs recognised by TFs were identified for all genes (Table S1A–E). The largest number of putative TF binding sites was found in the promoter region of *Os06g0565100* (1309 motifs), whereas the smallest number was observed in case of *Os02g0221900* (1023). When the numbers of TFs that may bind to the identified motifs were estimated, similar values in the range from 219 to 250 were obtained for all analysed promoters (Figure 4a). Among them, four, five, 14, eight and 31 TFs were specific only to the promoter sequence of *Os01g0700900*, *Os01g0701400*, *Os01g0701500*, *Os02g0221900*, *Os06g0565100*, respectively (Figure 4b). TFs common for all *MAX1* homologues were identified (60) (Table S2), as well as TFs common for different pairs of *MAX1* (Figure 1b). The biggest number of common TF binding sites was observed in the promoter regions of *Os02g0221900* and *Os06g0565100* (10) and *Os01g0700900* and *Os01g0701400* (8), whereas the lowest similarity in the TF binding sites was present between promoter regions of *Os01g0701500* and *Os02g0221900* (1) (Figure 4b).

### 3.2. Transcription Factors Specific to Os01g0700900

In the case of the promoter region of *Os01g0700900*, five motifs recognised by four different TFs were identified that are specific only to this one *MAX1* gene (Table 1; Table S3). For all of them the role in plant response to cold was previously described. Expression of *Os03g0820400* (TFmatrixID_0216) was induced by cold more than four-fold in rice cold tolerant cultivar Oro (da Maia) [51]; *Os10g0377300* (TFmatrixID_0298) was typed as candidate genes related to low-temperature tolerance [52]; whereas *Os12g0123700* (TFmatrixID_0381) and *Os02g0170300* (TFmatrixID_0529) were found to be cold-responsive in rice and *Oryza ofcinalis* [53]. Among other abiotic stresses, binding sites recognised by TFs involved in plant responses to drought [54,55,56,57], salt [58,59,60], arsenic [61], cadmium [62], nitrogen [63,64] or iron [65] deficiency were identified (Table 1; Table S3). Binding sites of TFs that play a role in plant response to pathogen attack, such as viruses [66], bacteria [67] or fungi [68], were also present. Finally, some of the TFs, that are specific only to *Os01g0700900* and are involved in developmental processes, such as root [69] or flower development [70,71], leaf senescence [72,73] and seed dormancy/germination [74] were found. Recently the function of *OsMADs57*, that exclusively bind the motif only in the promoter region of *Os01g0700900*, was described [75]. Knockdown of *OsMADs57* resulted in plant height reduction, inhibition of internode elongation and reduction in panicle exertion. Additionally, mutated plants contained less bioactive forms of gibberelins (GAs), when compared to wild-type, and were more sensitive to GA_3_ treatment [75]. This feature indicates the possible crosstalk between SL and GA biosynthesis pathways.

### 3.3. Transcription Factors Specific to Os01g0701400

Analysis of the *Os01g0701400* promoter region revealed five TF motifs that were not present in other *MAX1* homologues (Table 1; Table S4). TFs that belong to the TFmatrixID_0503 group were identified as regulators of flowering [71,76,77,78,79,80]. *Os02g0682200* (*OsMADS6*) and *Os04g0580700* (*OsMADS17*) are involved in the specification of floral organ identity, whereas *Os08g0531700* (*OsMADS7*) and *Os09g0507200* (*OsMADS8*) are involved in flower development [71]. Among other developmental processes, some of the identified TFs play a role in shaping the root architecture [81,82] (Table S4). A wide range of TFs that may bind the promoter of *Os01g0701400* were identified as related to abiotic stresses, including response to arsenic [61,83], cold [84], drought [55,56,57,85,86,87], phosphate deficiency [88] and submergence [89]. On the other hand, four genes that belong to the AP2 family (*Os03g0183200*, *Os07g0617000*, *Os09g0286600* and *Os09g0287000*), were previously described as induced by infection of *Xanthomonas oryzae* pv. *oryzae* [67]. Additionally two motifs, with unknown TFs were found: GLUTEBP2OS and ANAERO5CONSENSUS (Table S4). The first one regulates the transcription of genes encoding glutelin storage proteins [90], whereas the latter one was found in silico in promoters of 13 anaerobic genes involved in the fermentative pathway [91]. Finally, two TFs that may link SL biosynthesis with other phytohormones were identified. *OsERF67* (*Os07g0674800*) was significantly increased by ethylene and abscisic acid treatment, whereas treatment with auxin, GA or brassinosteroid did not affect the expression of *OsERF67*.

### 3.4. Transcription Factors Specific to Os01g0701500

In the promoter region of *Os01g0701500* the second highest number of specific TF binding motifs were found, however some TF groups (TFmatrixID_0386, TFmatrixID_0388, TFmatrixID_0389, TFmatrixID_0390, TFmatrixID_0395; as well as TFmatrixID_0524, TFmatrixID_0547) were represented by the same representatives (Table S5). Identified TFs, were involved in plant responses to various biotic stresses, including viruses [66], bacteria [92] and fungi [68] and different abiotic stresses (Table S5). Among TFs related to abiotic stresses, those involved in iron [65,93] and nitrogen deficiency [63,64,94], response to cold [95], drought [54,55,96,97], salt [58,60,98], cadmium [62], chromium [99], submergence [100] were found. Binding motifs for large number of TFs involved in developmental processes, including flower development [76,101,102,103,104,105,106], root development [81] and leaf senescence [107,108] (Table 1; Table S5) were identified in the promoter region of *Os01g0701500*. Additionally binding sites for TFs that are involved in secondary wall formation were identified [109,110] (Table S5). Two of them, *Os01g0701500* and *Os06g0131700*, are known as OsSWN1 and OsSWN2, respectively (SECONDARY WALL NAC DOMAIN PROTEIN1/2). It was shown that overexpression of *OsSWN1* strongly induced ectopic secondary wall formation [111]. Two other TFs, *Os06g0104200* and *Os08g0103900* were able to bind the promoter region of *AtMYB46* and functionally complement the *A. thaliana snd1/nst1* double mutant that exhibits lack of lignified secondary walls in fibres. Overexpression of *Os06g0104200* and *Os08g0103900* in *A. thaliana* causes ectopic deposition of cellulose, xylan and lignin [112]. Those results indicate that one of the *MAX1* homologues—*Os01g0701500* may be regulated by the group of TFs related to secondary wall formation.

### 3.5. Transcription Factors Specific to Os02g0221900

In the promoter region of *Os02g0221900*, motifs recognised by five different TFs, that are specific only for this one *MAX1* gene, were identified (Table 1; Table S6). Interestingly none of the TFs that were involved in plant response to biotic stresses had been found. In the aspect of TFs related to abiotic stresses, those involved in response to cold [113], drought [114] and chromium [99] were identified. Whereas among TFs related to developmental processes, those involved in root development [81,115], flower development [116,117] were able to bind promoter of *Os02g0221900*. Also the motif that is recognised by *OsVP1* (*Os01g0911700*), rice orthologue of Arabidopsis ABI3, is present in the promoter of *Os02g0221900*. Experimental data indicate that *OsVP1* is a major determinant of seed specificity and regulate the spatial pattern of expression of genes in developing seed [118]. Additionally only this one *MAX1* homolog is under control of *OsTCP5* (*Os01g0763200*), a TF that belongs to the cell division-regulating TCP family, that is regulated by SL and CK [6]. It was proved that *OsTCP5* is involved in SL-controlled axillary bud outgrowths [119] and in the control of cell division in rice mesocotyl, that depends on SL and CK [6].

### 3.6. Transcription Factors Specific to Os06g0565100

The highest number of TF-binding sites were found in the promoter region of *Os06g0565100* (1309) (Table S1) and also the highest number of TFs specific only for this MAX1 homolog was identified (Table S7). Similar to the previous genes that were analysed, *Os06g0565100* may be also under control of TFs involved in plant response to bacteria [120,121], fungi [67,68,122,123,124] and insects [125,126], as well as cold [52,53,84,127,128], drought [54,55,56,57,86,87,114], cadmium [62,129], submergence [91,130,131], salt [85,132], arsenic [61,83], or iron [133], nitrogen [63,64,94,134] and phosphorus [88] deficiency. In the promoter region of *Os06g0565100* motifs recognised by TFs are related to flower [116,135] and root [81,115,136] development, or controlling shoot architecture [137]. Among TFs that are able to bind the promoter of *Os06g0565100*, four ethylene response TFs were found: *Os02g0202000* (OsWR1), *Os06g0604000* (OsWR2), *Os02g0797100* (OsWR3) and *Os06g0181700* (OsWR4). Functional analysis of OsWR1 indicated that this TF is a positive regulator of drought resistance in rice, because it promotes expression of genes that are involved in wax synthesis, and therefore involved in water loss reduction [138]. Additionally it was shown that transcript levels of *OsWR*1 were induced by drought, salt and ABA treatment [138]. On the other hand OsAP2-39 (Os04g0610400), that may recognise ten motifs in the promoter region of *Os06g0565100*, was described as a key regulator of the interaction between ABA and GAs in rice [139]. Overexpression of *OsAP2-39* results in yield reduction by decreasing the number of seeds and upregulation of ABA biosynthesis via increased activity of *OsNCED-I* gene (encoding 9-cis-epoxycarotenoid dioxygenase) involved in this process. Finally, overexpression of *OsAP2-39* upregulates the expression of the ELONGATION OF UPPER MOST INTERNODE I (EUI) gene that is involved in the epoxidation of the active GAs, and thus reduces the level of bioactive GAs in rice [139].

### 3.7. MiRNAs That May Bind Rice MAX1 Homologues

**Figure 5:**
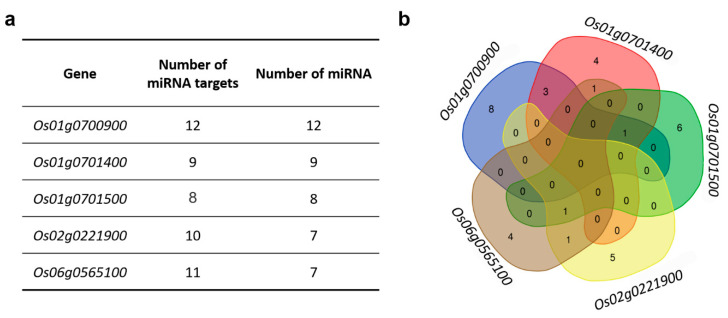
MiRNAs that recognise targets that are present in sequence of rice *MAX1* genes. (**a**) Number of miRNA targets found in *MAX1* homologs and number of miRNA that target sequences of *MAX1s*. (**b**) The Venn diagram illustrates miRNA that are specific to each of rice *MAX1* genes and those that are common for different *MAX1* homologues.

The highest number of miRNA targets was identified for *Os01g0700900*—12, for the other *MAX1s* the number of miRNA target sites ranged from eight to 11 (Figure 5a, Table S8). In the case of miRNA targets identified for *Os01g0700900, Os01g0701400* and *Os01g0701500*, each target site was recognised by various miRNAs. In the sequence of *Os02g0221900* four target sites for osa-miR5075 were found, whereas for in the sequence of *Os06g05651000*, two targets for osa-miR2927, osa-miR5075, osa-miR3980a-3p and osa-miR3980b-3p were identified (Table S8). Among all identified miRNAs, eight were specific only to *Os01g0700900*, six for *Os01g0701500*, five for *Os02g0221900* and four for *Os01g0701400* and *Os06g05651000* genes (Figure 5b). None of the miRNAs identified match four or all five rice *MAX1* genes. Only two miRNAs match the motifs in three *MAX1* homologues: osa-miR419 (*Os01g0700900, Os01g0701400, Os01g0701500*) and osa-miR5075 (*Os01g0701500, Os02g0221900, Os06g0565100*) (Table S8).

### 3.8. MiRNA Specific to Os01g0700900

Within the miRNAs identified for *Os01g0700900* only eight were specific to this one *MAX1* gene. Within these miRNAs, *osa-miR2055*, which is expressed preferentially in rice roots, plays the role in plant response to increased temperature (Table 2). When two rice varieties were compared: tolerant to heat (Nagina 22) and susceptible (Vandana), upregulation of *osa-miR2055* was observed in the heat-tolerant variety in response to short (42 °C/36 °C day/night for 24 h) and long (42 °C/36 °C day/night for 5 days) heat treatment [140]. A second miRNA, specific for *Os01g0700900* was *osa-miR1432-3p* (Table S8), which is known to be involved in rice response to rice stripe virus (Table 2), because targets of that miRNA are well characterised disease resistance genes (*LOC_Os02g42160* and *LOC_Os07g35680*) encoding wall-associated receptor kinase-like 1 and cysteine-rich receptor-like protein kinase 8, respectively [141].

### 3.9. MiRNA Specific to Os01g0701400

Only four miRNAs (within nine identified) were specific to *Os01g0701400* (Figure 5a). Within these miRNA’s one was involved in abiotic stress response [136], two in biotic stress response [137,138] and one in developmental process [139,140]. Among abiotic stresses, response to heat was described for *osa-miR166d-5p* (Table 2), however in contrast to the previously described miRNA specific to *Os01g0700900* (osa-miR2055), in that case expression of *osa-miR166d-5p* was inhibited during heat treatment in tolerant variety Nagina 22 [136]. On the other hand, expression of *osa-miR166d-5p* increased more than 36-fold, in response to rice stripe virus infection [137]. Also, when rice plants were infected by southern rice black-streaked dwarf virus, the expression of another miRNA increased, *osa-miR2097-3p*, which was specific only to this *MAX1* gene [138]. Those experimental data suggest that both miRNAs (*osa-miR166d-5p* and *osa-miR2097-3p*) are involved in plant resistance to viruses (Table 2). Finally, expression of *osa-miR5514* was two-fold higher in pollen (during meiosis) of diploid rice, when compared with autotetraploid [140], this feature suggests the role of *osa-miR5514* in pollen development (Table 2).

### 3.10. MiRNA Specific to Os01g0701500

Within the eight miRNAs identified for *Os01g0701500*, six were specific to this *MAX1* gene. Within these miRNAs, two were associated with responses to biotic stresses: bacteria *(osa-miR166b-5p*) [141] and fungi (*osa-miR2103*) infections [142] (Table 2). A wide range of abiotic stresses were represented by identified miRNAs, including plant responses to heat: *osa-miR166b-5p*, *osa-miR528-5p* [136] and *osa-miR5519* [143], drought: *osa-miR528-5p* [144], cold: *osa-miR528-5p* [145] and zinc deficiency: *osa-miR528-5p* [146]. Experimental data indicated that a single miRNA, *osa-miR528-5p*, was upregulated when rice plants were exposed to zinc starvation [146], but was also upregulated over four-fold by cold temperature stress in cold-tolerant rice variety (Hitomebore) [145], and additionally plays the role in drought response (Table 2). *Osa-miR528-5p* showed the opposite differential expression pattern between drought-tolerant (Vandana, Aday Sel) and drought-susceptible (IR64) rice varieties. In leaf tissues, it was down-regulated in drought-tolerant rice varieties but up-regulated in drought-susceptible rice variety. What is also important is that the expression level of *osa-miR528-5p* was significantly higher in drought-tolerant rice varieties compared to a drought-susceptible rice variety under control conditions [144]. The explanation of this wide range of stress responses in which *miR528-5p* is involved, might be the fact that this miRNA is ABA-responsive (Table 2). It was shown that in the tissue of rice ABA-deficient mutant *Osaba1*, the expression of *miR528-5p* is significantly upregulated (almost five-fold) when compared to the wild-type plants [147]. Finally, *osa-miR528-5p* was characterised as involved in flower development in rice [140] (Table 2). Studies carried out on di- and autotetraploid rice revealed that *osa-miR528-5p* plays a role in the early stage of pollen development through silencing the expression of the target genes (i.e., LOC_Os06g06050), and therefore resulted in abnormal pollen development in autotetraploid rice [140].

### 3.11. MiRNA Specific to Os02g0221900

Within miRNAs identified for *Os02g0221900*, five were specific to this one *MAX1* gene. Within these miRNAs one (*osa-miR1430*) was functionally characterised. It was proved that *osa-miR1430* and *osa-miR169* may target the same gene encoding a nuclear transcription factor Y subunit (*LOC_Os12g42400*) [142]. Since members of *osa-miR169* were upregulated in the *phyB* mutant [142], *osa-miR1430* may also be involved in phytochromeB-mediated light signalling pathway (Table 2).

### 3.12. MiRNA Specific for Os06g0565100

Within the seven miRNAs identified for *Os06g0565100* only four were specific for this one *MAX1* gene. *Osa-miR827* is a well-known regulator of rice responses to phosphorus deficiency [143,144]. Under phosphorus starvation the accumulation of *osa-miR827* was observed in both shoots and roots, however stronger expression was noted in shoots [143,144]. For the second miRNA that was specific only for *Os06g0565100*, four possible functions were proposed: involved in response to heat [140], wax synthesis [145], leaf senescence [146] and crosstalk with brassinosteroids [147] (Table 2). In the case of heat tolerance, expression of *osa-miR1848* was observed only in the tissues of a heat-tolerance variety during exposition to the high temperature [140]. Analyses of expression patterns in leaves of two super hybrid rice, Nei-2-You 6 (N2Y6, age-resistant rice) and LiangYou-Pei 9 (LYP9, age-sensitive rice) revealed that *osa-miR1848* is involved in leaf senescence via targeting NAC TFs [146]. On the other hand the known target of *osa-miR1848* is *OsWS1* (*Oryza sativa wax synthase isoform 1*) encoding a protein involved in cuticular wax formation [145]. It was shown that in the leaves of *osa-miR1848*-overexpressing plants, *OsWS1* expression decreased approximately five-fold compared with wild type plants [145]. Finally, the obtusifoliol 14α-demethylase gene *OsCYP51G3* was described as a target of *osa-miR1848*. OsCYP51G3 is involved in phytosterol biosynthesis, which is a precursor of brassinosteroids. Thus, overexpression of *osa-miR1848* reduced brassinosteoids biosynthesis, because the relative amounts of six brassinosteroids (teasterone, 3-dehydroteasterone, typhasterol, 6-deoxoteasterone, 6-deoxotyphasterol and castasterone) decreased in plants overexpressing *osa-miR1848*, when compared with wild-type plants [147].

### 3.13. Profile Expression of MAX1 Homologues

**Figure 6:**
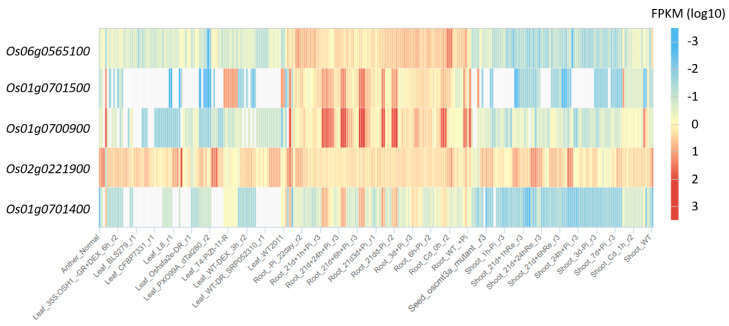
Heat map chart of rice *MAX1* gene expression profiles. FPKM—the number of fragments aligned per kilobases of the transcript per million mappable fragments from the total dataset. Description of the experiments was given in Table S9. White spots represent lack of data in this specific experiment to a given gene, each line corresponds to the single repetition.

Gene expression data of Nipponbare rice variety from 284 experiments, carried out on plants at different age (from 3-days-old seedling, up to flowering time), different organs (shoot, root, leaf, panicle, anther, callus, seeds) and different treatment (i.e., drought, phosphorus starvation or cadmium treatment) were analysed (Figure 6, Table S9). In the case of 181 experiments, expression data were available for all five rice *MAX1* genes. In the case of 35 experiments the pattern of expression of all five *MAX1* homologues was similar (Table S9). On the other hand, in 61% of the experiments (111 from 181) the expression of one *MAX1* gene was different in comparison to the other four *MAX1* homologues (Table S9). On the other hand, in mature seeds, induced expression was observed only for *Os01g0701500*, whereas expression of others *MAX1s* was repressed (project ID SRP028376). Similar results were obtained for leaf tissue culture (Project ID SRP017256). Whereas in anthers, after and before flowering, only the expression of *Os02g0221900* was upregulated (project ID SRP047482 and DRP001762). Other experiments when the expression profile of the single *MAX1* was different in comparison to the other four *MAX1* homologues, specific time points of phosphorus starvation were carried out, in both roots and shoots of rice plants at different ages (Table S9).

### 3.14. Co-Expression Gene Networks of MAX1 Homologues

**Figure 7:**
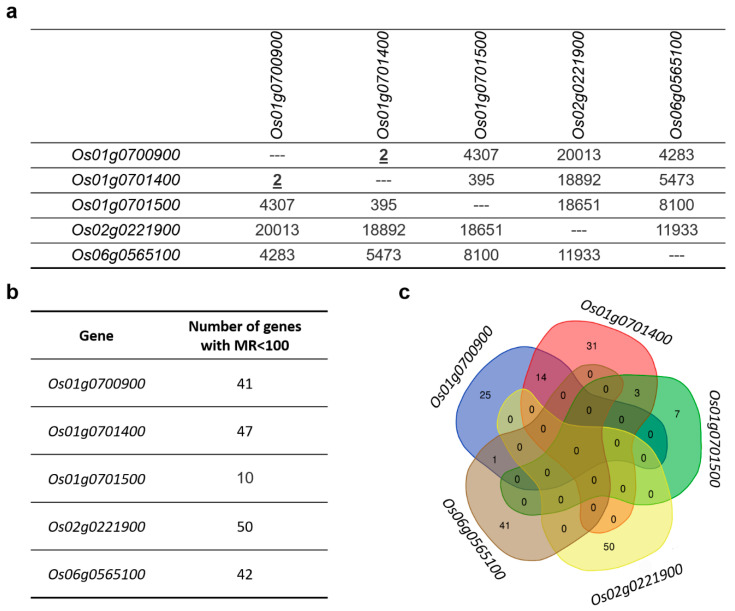
Co-expression gene network (CGN) of *MAX1* genes. (**a**) Mutual Rank (MR) of co-expression between *MAX1s.* (**b**) Number of genes that exhibited similar expression pattern, such as *MAX1* homologues (genes with MR lower than 100). (**c**) The Venn diagram illustrates gens from CGN that are specific to each of rice *MAX1* genes and those that are common for different *MAX1* homologues.

For each of the *MAX1* homologues, a list of 27,201 co-expressed genes was obtained from the Rice Expression Database [48], that collects data from the different RNA-Seq experiments (Table S10). The two *MAX1* homologues, *Os01g0700900* and *Os01g0701400*, demonstrated strong co-expression and were listed on the first positions of their co-expression gene network (CGN) list (Figure 7a, Table S10). These two are also phylogenetically closely related (Figure 3) and exhibited the highest number of shared miRNA motifs among *MAX1* genes (Figure 5b). No other *MAX1* homologs reach the criteria of MR lower then 100 (Figure 7a). In the case of four *MAX1* homologs (*Os01g0700900*, *Os01g0701400*, *Os02g0221900*, *Os06g0565100*) more than 40 genes in CGN with MR lower than 100 were identified. Whereas in the case of *Os01g0701500* only 10 genes met these requirements (Figure 7b). Consistent with the high co-expression pattern of *Os01g0700900* and *Os01g0701400*, the highest number (14) of common genes was present in the CGNs of both *MAX1s.* No common genes from CGN with MR lower than 100 were found between *Os01g0701500, Os02g0221900* and *Os06g0565100* (Figure 7c).

### 3.15. Co-Expression Gene Network of Os01g0700900

The analysis revealed that 25 genes were highly co-expressed (MR lower then 100) exclusively with *Os01g0700900*. Three genes from CGN of *Os01g0700900*, were described as involved in rice response to cadmium: *Os08g0106300* (MR = 16.793, 2nd position in list of co-expressed genes) [148], *Os08g0189200* (MR = 91.913) [149] and *Os05g0162000* (MR = 94.106) [129] (Table 3). Moreover, gene ontology (GO) analysis also revealed the gene *Os04g0400800* (MR = 16.793, 3rd position in list of co-expressed genes) that encodes a protein that contains a heavy metal transport/detoxification domain (Table S10). Finally, *Os03g0149000* (MR = 99.287), *Os06g0185500* (MR = 80.833) and *Os12g0454800* (MR = 85.229) play a role in rice response to ferulic acid [150], copper [151] and chromium [99], respectively. Changed expression of *Os05g0162000* that encodes a peroxidase, was observed not only in case of the rice response to cadmium, but also in the case of exposure to an excess of iron [133] and defence against rice blast fungus *Magnaporthe oryzae* [152] (Table 3). Another gene from *Os01g0700900* CGN: *Os03g0368900* (MR = 85.229) encoding a peroxidase precursor is involved in ROS homeostasis in rice and is under the control of MADS3 (Os01g0201700) that regulates late anther development and pollen formation [153]. Finally, *Os10g0390800* (MR = 94.106), encoding another peroxidase (Table S10), plays the role in rice response to drought [56], phosphorus starvation [88] and bacterial infection [92] (Table 3).

### 3.16. Co-Expression Gene Network of Os01g0701400

In the CGN of *Os01g0701400* (MR < 100) 31 genes were specific only to this *MAX1* homologue (Figure 7c). One of the identified genes, *Os07g0531400* (MR = 72.595), was described in three independent experiments as a component of rice in response to deficiency or excess of iron [65,133,154], similarly to *Os03g0368300* (MR = 39.987) [65] (Table 3). Also, genes involved in response to nitrogen status, *Os05g0246300* (MR = 75.736) [155], *Os02g0143400* (MR = 82.432) [64] and *Os06g0104900* (MR = 75.736) [156] were found in the CGN of *Os01g0701400* (Table 3). Two other genes, *Os02g0754400* (MR = 95.812) and *Os10g0191300* (MR = 81.425) play a role in response to arsenic [83] and silicon [157], respectively (Table 3). However, it was postulated that, *Os10g0191300*, which encodes a pathogenesis-related protein (Table S10), might be involved in rice defence against insects [158] and fungi [157]. Other pathogen resistance genes are *Os03g0760500* (MR = 96.156)—against bacteria [120], and *Os10g0335000* (MR = 81.994)—against fungi [159]. A group of genes related to rice in response to abiotic stresses was found among CGN of *Os01g0701400*, including salinity (*Os10g0191300*, MR = 81.425) [160], submergence (*Os04g0521100*, MR = 38.21) [161], drought (*Os07g0646800*, MR = 77.421) [57] as well as wounding (*Os10g0191300*, MR = 81.994) [162] (Table 3).

Another gene, *Os07g0174900* (MR = 81.994), was strongly expressed in the outer part of roots, and thus could be involved in suberin and lignin biosynthesis [163]. Whereas according to the UniProt Database three additional genes are related with cell wall formation, *Os05g0246300*, *Os10g0335000* and *Os03g0368000* (MR = 20.881) (UniProt). *Os01g0701400* was expressed during anther development and pollen formation (*Os03g0368000*) [153]. Whereas high ABA-induced expression of *Os12g0455000* (MR = 20.881) was observed during root hair elongation [164]. Finally, analysis of rice lines with overexpression of *Os02g0662000* (MR = 71.498), which encodes Rcc3, a proline-rich protein (PRP), revealed that Os02g0662000 promotes development of the root system via increased accumulation of auxin in the root, and additionally increased tolerance to salt stress [165].

### 3.17. Co-Expression Gene Network of Os01g0701500

Seven (from 10) genes found in CGN of *Os01g0701500* that met the criteria MR < 100 were specific to this *MAX1* homolog. Among them, a function is described in literature for only two. The first one is: *Os06g0221000* (MR = 33.764), which encodes one of the TF belongs to MYB family (MYB-81), and its expression was upregulated by mercury (25 µM) treatment [166], and the second one—*Os04g0590100* (MR = 35.567) was involved in rice responses to cadmium [149]. The protein encoded by this gene contains a heavy metal transport/detoxification domain (Table S10).

### 3.18. Co-Expression Gene Network of Os02g0221900

All 50 genes from CGN of *Os02g0221900* with MR < 100 were specific only for this one *MAX1* homologue (Figure 7a). Among that number, experimental data of the possible functions were available for 14 genes (Table S10). The largest group of genes was involved in response to biotic stresses, including fungi (*Os01g0701700*, MR = 41.952 [167], *Os04g0637000*, MR = 15.1, [168], *Os01g0117200*, MR = 57.35, [169]; *Os05g0409500*, MR = 64.312 [170]; *Os03g0798200*, MR = 90.111, [171]), bacteria (*Os05g0365300*, MR = 89.778, [172], *Os04g0637000* [173]) and insects (*Os04g0637000* [168]) (Table S3, Table S10). A wide range of abiotic stresses was represented in CGN of *Os02g0221900*, including response to cold (*Os01g0701700* [127]; *Os05g0409500* [128]), drought (*Os05g0557700* [174], *Os05g0409500*, [57]), phosphorus status (*Os03g0348200*, MR = 50.279, [175]; *Os04g0650700,* MR = 20, [175]; *Os05g0557700* [176,177]; *Os03g0647600*, MR = 74.162 [178], *Os01g0882800*, MR = 98.122 [64]), nitrogen status (*Os01g0870300*, MR = 90.427, [179]; *Os01g0882800* [180]) and silicone (*Os05g0409500*, [157]) (Table 3, Table S10). On the other hand, *Os10g0463400* (MR = 61.425), encoding a B-type response regulator (Edh1, Early headingdate1), is involved in rice flowering. It was shown that the loss of function of Edh1 led to prolongation of the vegetative growth [181]. Whereas *OsASNase2* (*Os04g0650700*), encoding asparaginase2, is involved in asparagine catabolism, and plays a role in the development of rice grains. *OsASNase2* is the major asparaginase isoform in rice shoots, and is expressed in the dorsal vascular bundles and in developing grains, as well as in mesophyll and phloem companion cells of senescent flag leaves [182]. Finally, two genes (*Os12g0618600*, MR = 67.149 and *Os03g0647600*), were described as targets of ABA-responsive miRNA in rice [183], whereas *Os04g0637000* encoding TF OsTGAP1, is induced under jasmonic acid treatment, and plays a role in biosynthesis of diterpenoid phytoalexin [184].

### 3.19. Co-Expression Gene Network of Os06g0565100

Among 42 genes from *Os06g0565100* CGN (MR < 100), 41 were specific only to this *MAX1* homologue (Figure 7c), and for them, 14 functions were proposed, based on the available literature (Table 3, Table S10). Again, the group of genes related to abiotic stresses was the largest. Expression of one of the genes (*Os01g0974200*, MR = 63.718) encoding metallothionein (OsMT2c) was induced by cadmium, nickel, magnesium, copper and cold [129,185], whereas *Os02g0745100* (MR = 28.142) plays a role in response to arsenic [83,186,187], mercury [166] and heat [188] (Table 3). Also, genes involved in responses to phosphorus status (*Os03g0223400*, MR = 46.174, [180]) and nitrogen status (*Os12g0586300*, MR = 44.125 [189]; *Os03g0223400* [156]; *Os05g0447200*, MR = 51.827 [190]), drought (*Os10g0567900*, MR = 96.343, [191]) and salt stress (*Os01g0756700*, MR = 34.467 [192]; *Os02g0820900*, MR = 31.177) (Table 3) were identified. *Os01g0756700* that enhances salt tolerance in rice, encodes Shaker family K^+^ channel KAT1 (OsKAT1). The expression of *OsKAT1* was restricted to internodes and rachides of wild-type rice, whereas other Shaker family genes were expressed in various organs [192]. Some of the genes from CGN of *Os06g0565100* were also involved in rice response to pathogen attack, such as bacteria (*Os10g0567900* [172]), insects (*Os01g0974200* [193]) and fungi (*Os12g0586300* [67], *Os03g0854600*, MR = 33.764 [194]). Finally, genes related to developmental processes were identified in CGN of analysed *MAX1*. *Os01g0710200* (MR = 88.978) that encodes polyamine oxidase7 (OsPAO7) is specifically expressed in anthers, with an expressional peak at the bicellular pollen stage [195]. On the other hand, *Os03g0223400* (MR = 46.174), encoding glutamine synthetase (OsGS1;2), is involved in the development of rice grains [182,196] and glutamine-dependent tiller outgrowth [197]. Another gene, *Os01g0126100* (MR = 21.977), was described as a target of ABA-responsive miRNA [183]. Whereas expression of *Os08g0167000* (MR = 13.711), which encodes one of the half-size adenosine triphosphate-binding cassette transporter subgroup G (ABCG), was induced by salicid acid and repressed by auxin treatment [198]. Finally, *Os05g0447200* is *AUX2*, one of the auxin-responsive genes, and was highly induced under microgravity conditions [199].

### 3.20. Gene Ontology for Genes Co-Expressed with MAX1s

**Figure 8:**
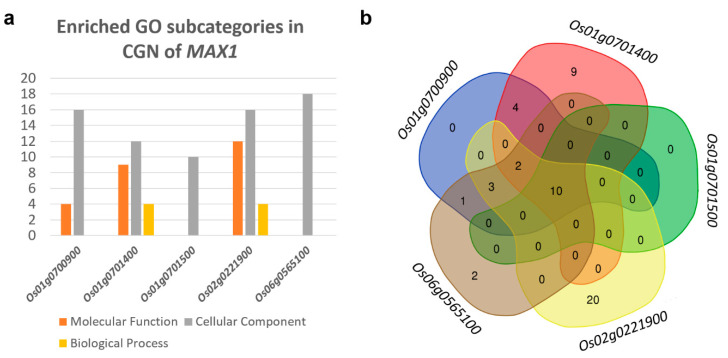
Co-expression gene network (CGN) of *MAX1* genes. (**a**) Mutual Rank (MR) of co-expression between *MAX1s.* (**b**) Number of genes that exhibited similar expression pattern, such as *MAX1* homologues (genes with MR lower than 100). (**c**) The Venn diagram illustrates genes from CGN that are specific to each rice *MAX1* gene and those that are common for different *MAX1* homologues.

Previous analyses relating to the function of genes from CGNs of *MAX1s*, were based on the information obtained using experimental approaches. Here, we additionally check the gene ontology (GO) for all genes that were co-expressed with each *MAX1* (MR < 100). The obtained results revealed a wide range of ontologies in all three categories: molecular function (F), biological process (P) and cellular component (C) for each of CGNs (Table S11A–E). Enrichment analysis allowed us to identify the GO that were overrepresented in the CGN of each *MAX1* gene (Table S12). For CGN of *Os01g0700900* (36 genes) 20 enriched GOs were found that represent two subcategories: F (4) and C (16). In the CGN of *Os01g0701400* (44 genes) all three subgroups of enriched GO were identified: F (9), C (12) and P (4). Among genes from *Os01g0701500* CGN (10 genes) and *Os06g0565100* CGN (36 genes) only GOs from the C subcategory were identified, 10 and 18, respectively. Analysis of *Os02g0221900* CGN (40 genes) revealed the highest number of GOs (35) that represent all three subgroups: F (12), C (16) and P (7) (Figure 8a). Comparative analysis of enriched GOs revealed that some of them are specific only for genes from the CGN of *Os01g0701400* (9 GOs), *Os06g0565100* (2 GOs) and *Os02g0221900* (20 GOs) (Figure 8b).

## 4. Discussion

The number of developmental processes and abiotic/biotic stresses that are SL-dependent are rapidly being uncovered [13]. The majority of our knowledge about the role of SL in plants comes from the analysis of mutants disturbed in SL biosynthesis and signalling. In the case of the biosynthesis mutants, plants with disorders in core biosynthesis pathway are usually used, because inactivation of the enzymes involved in initial stages of SL production results in lack/decreased amount of all SLs in plants [33]. However, each plant produces different SLs, and the composition of produced SL mixture may vary because of developmental stage or environmental conditions [200]. Moreover new SLs are still being discovered [15], and the question why plants are producing different classes of SLs is still open. Up to now, the origins of structural diversity of SLs remains unknown. The core of SL biosynthesis (up to CL) is highly conserved in mono- and dicots, whereas the next stages that result in the formation of various SLs, are different [23]. In *A. thaliana* MAX1 catalyses the oxidation of CL to CLA [19], which is next converted into an unknown SL-like product [33]. On the other hand, in rice there are five *MAX1* homologues*,* and it was postulated that four of them might be involved in different steps of SL biosynthesis [35]. However, the functions of the different produced SLs have not been fully reported so far, and specific enzymes involved in their biosynthesis have not been described yet.

Previously we have analysed rice and *A. thaliana* genes encoding proteins involved in SL biosynthesis, which allowed us to propose the new functions of SLs, and to describe mechanisms that may regulate their expression [11]. So far, various research reports that confirmed our predictions were published (reviewed by [12,201]). Here, we focused on all five rice *MAX1* genes. Our in silico analyses revealed the set of TFs and miRNAs that may be involved in the regulation of *MAX1* homologues in rice. Finally, analysis of RNA-seq data was used to describe the profiles of *MAX1* expression and reveal the genes from their co-expression gene networks. All the data indicate that there are specific mechanisms that regulate the expression of each single *MAX1*, and additionally, processes specific for individual *MAX1s* can be proposed.

### 4.1. TFs That Regulate Expression of MAX1 Homologues

Because TFs are able to regulate the spatial expression of the many different genes, TFs play a crucial role in the coordination of plant growth, development and plant responses to different stresses [202]. More than 200 TFs that recognise motifs in the promoters of single *MAX1* were identified (Figure 4). Among them almost 25% (60 motifs) were common for all *MAX1s,* which indicates that in many cases expression of all five homologues exhibits the same regulation mechanisms (Figure 4b). Despite this, it was possible to select TF-binding sites for each of the *MAX1* homologues that were specific only to a single *MAX1* gene. It should be noted that binding sites might be recognised by different TFs that belongs to the same family (Tables S3–S6). Thus, the expression of each of the *MAX1* homologues could be induced or repressed under specific regulatory mechanisms. Analysis of the TFs that were specific to single *MAX1s* revealed that some of them were already functionally characterised (Tables S3–S6) (Table 1). For example, one of the *MAX1* homologues (*Os01g0701500*) is under control of two TFs that act as master regulators of secondary wall formation in rice: SECONDARY WALL NAC DOMAIN PROTEIN1 and 2 (OsSWN1, Os01g0701500 and OsSWN2, Os06g0131700). It was shown that *OsSWN1* and *OsSWN2* are expressed in cells where secondary cell walls are formed and can alter secondary cell wall formation in rice: *OsSWN1* is highly active in sclerenchymatous cells of the leaf blade and less active in xylem cells, whereas *OsSWN2* is highly active in xylem cells and less active in sclerenchymatous cells [111]. Additionally, two other TFs (OsSWN3, Os08g0103900 and OsSWN7, Os06g0104200) that are involved in cell wall biosynthesis, also bind this *MAX1* exclusively. Zhong and co-workers demonstrated that overexpression of rice *OsSWN1, OsSWN3* and *OsSWN7* genes in *A. thaliana* induces ectopic deposition of cellulose, xylan and lignin in secondary walls. These data indicated that the abovementioned three TFs are important players in secondary wall formation in rice [112] and all of them regulate expression of single *MAX1* gene (Table 3). Thus, it might be speculated that this *MAX1* homolog (*Os01g0701500*) is involved in secondary wall formation in rice. On the other hand, another *MAX1* homologue (*Os02g0221900*) is under regulation of one of the major determinant of the seed specificity—OsVP1 (Os01g0911700). Analysis of temporal and spatial expression patterns of the *OsVP1*, revealed that activity of this TF could be detected in embryos as early as 2–3 days after pollination (DAP) and thereafter became preferentially localised to shoot, radicle and vascular tissues during the embryo development at both the mRNA and protein levels. Whereas in the aleurone layers, OsVP1 mRNA and protein were detected after 6 DAP [118]. It was also shown that OsVP1 is required for the induction of ABA-regulated genes that include genes encoding the late embryogenesis abundant (lea) protein [203]. Based on these results the role of another single *MAX1* homologue (*Os02g0221900*) during rice seed formation and development can be postulated. Both described functions, secondary wall formation and seed development were not previously reported for SLs. This might be because there are ‘non-canonical’ SL functions—the functions in which specific SLs, produced by a single *MAX1*, are involved, and the respective single mutants have not yet been analysed.

It also should be noted that TFs that exclusively bind the promoter region of a single *MAX1* may be also involved in the same processes as the TFs identified for other *MAX1* homologues. However, the presence of the unique TFs motifs in the promoter region of different *MAX1s* indicates that, under specific conditions, their expression will be regulated by different mechanisms. Lastly, it should be emphasised that our in silico analyses need to be confirmed by DNA–protein interaction experiments.

### 4.2. Rice MAX1s Are Regulated by Different miRNAs

MicroRNAs (miRNAs) are short, non-coding RNAs that identify complementary sites in mRNAs and target selected mRNAs for repression [204]. In contrast to the TFs, miRNAs were unique for each of the *MAX1*. For each of *MAX1*, at least eight miRNAs were identified, and what is more important, at least four of them were specific only to the mRNA of single *MAX1* (Figure 5). Moreover, none of the identified miRNAs were able to bind four or all five rice *MAX1* genes. Only two miRNAs recognise motifs in three *MAX1* homologues: *osa-miR419* (*Os01g0700900*, *Os01g0701400*, *Os01g0701500*) and *osa-miR5075* (*Os01g0701500*, *Os02g0221900*, *Os06g0565100*) (Table S8). Based on the known function of the miRNAs that may regulate *MAX1*, processes can be postulated that may involve specific *MAX1s*. For example, the mRNA of *Os01g0701500* is matched by *osa-miR528-5p*, which is involved in a wide range of developmental processes and stress responses (Table 2). Analysis of the rice ABA-deficient mutant, *Osaba1*, revealed a five-fold increase in expression of *osa-miR528-5p* compared to the wild-type [183]. Thus, an ABA-dependent miRNA may regulate the expression of gene-encoding enzyme involved in SL biosynthesis. Recently, Visentin et al., described that exogenous application of SL results in the accumulation of *miR156* in tomato, and moreover, an increase in guard cell sensitivity to ABA and stomatal closure was observed [205]. Here, we find out that an ABA-dependent miRNA may regulate one of the SL biosynthesis genes. This feature may indicate further crosstalk between SLs and ABA.

On the other hand, a second *MAX1* homologue—*Os06g0565100*—may be under the regulation of *osa-miR1848* (Table 2). This miRNA plays a role in various processes including wax biosynthesis. This observation is in agreement with results obtained for TFs that regulate expression of *Os06g0565100*, including OsWR1, which promotes the expression of genes involved in wax synthesis. There is increasing evidence that SLs are involved in plant response to drought [205,206,207] and one of the components of plant responses to drought is deposition of waxes. Thus, it can be proposed that one of the MAX1 homologue plays a role in wax biosynthesis and deposition, which may help plants to adapt to drought conditions.

### 4.3. Expression Profiles of MAX1s and Their Co-Expression Gene Networks

Analysis of RNA-seq data revealed that induced expression of *MAX1* genes was observed when rice plants were exposed to nitrogen deficiency. However, this induction was mainly observed in roots for four *MAX1* homologues: *Os01g0700900*, *Os01g0701500*, *Os02g0221900* and *Os06g0565100*. Whereas, under the same conditions, the expression of *Os01g0701400* in root tissues decreased (Table S9). On the other hand, some of experimental data regarding gene expression confirmed our in silico prediction regarding specific roles of single *MAX1* genes. For example, only *Os02g0221900* expression was upregulated in seeds, whereas the transcription of all other *MAX1s* was downregulated (Table S9). Previously we showed that *Os02g0221900* is under regulation of the main seed-specific TF (OsVP1). RNA-seq data confirmed that only this *MAX1* is highly active during seed development. This strengthens our postulate that the regulation of seed development could be a non-canonical SL function in plants, which is mediated by *Os02g0221900.*

Analysis of genes that are co-expressed with individual *MAX1s* revealed that each *MAX1* homologue belongs to its own CGN that contains a number of genes specific to that *MAX1*. Interestingly, only in the CGN of *Os01g0700900* and *Os01g0701500* (when MR < 100) the other *MAX1* (*Os01g0701500* and *Os01g0700900*) was found (Figure 7). The data confirmed that *MAX1* genes are likely controlled by different regulatory mechanisms. Investigation of the function of genes that are co-expressed with *MAX1s* suggest that *Os01g0700900*, for example, could be involved in rice responses to various heavy metals (such as cadmium, chromium, copper), whereas other *MAX1s* are co-expressed with genes involved in responses to arsenic (*Os01g0701400*), cadmium and mercury (*Os01g0701500*), or arsenic, cadmium, copper, magnesium, mercury and nickel (*Os06g0565100*). Thus, it can be speculated that SLs play a role in plant responses to heavy metals that were not previously reported. Incidentally, the Bala rice cultivar (deletion of *Os01g0700900* and *Os01g0701400* on chromosome 1) expresses susceptibility to germanium toxicity [208]. Four of the five *MAX1s* were also co-expressed with genes that are related to pathogen (bacteria, fungi, insects) attack. That role of SLs was previously proposed [11] and some experimental data in this field were also published (reviewed by [12]). On the other hand, in the CGNs of four *MAX1* homologs, genes involved in flower development were also found, including, for example, a regulator of flowering (*Edh1*, *Early headingdate1*) and an enzyme specific for anthers and developing pollens (*OsPAO7*, *polyamine oxidase7*). Thus, new canonical functions of SLs that relate to flower development or responses to heavy metals can be postulated.

## 5. Conclusions

Based on in silico analysis and the analysis of RNA-seq experiments we suggest that the five *MAX1* homologues could be involved in different developmental processes and stress responses in rice. The predicted ‘non-SL-canonical’ functions in plants can now be confirmed by analysis of mutants in single *MAX1* genes. MAX1s are involved in the late stages of SL biosynthesis and they are responsible for much of the structural diversity of SLs. Thus, the prediction is that the products of MAX1 activity will play a role in different process. Here, we describe mechanisms that may regulate transcription and post-transcriptional regulation of each of the *MAX1* homologues in rice. The obtained data indicate the presence of regulatory mechanisms that are specific to single *MAX1s*, and also highlight candidates (TFs and miRNA) for further investigation about the role of single *MAX1* genes. Finally, analysis of *MAX1* expression profiles and genes that are co-expressed with *MAX1* homologues in rice provide some experimental support to our in silico predictions. Based on those data, new ‘SL-canonical’ functions in rice can be postulated, such as the regulation of flower development or responses to heavy metals. On the other hand, the presented data allows us to also suggest some of ‘non-SL-canonical’ functions related to single *MAX1* homologues (products of the enzymatic activity of single MAX1s), such as wax biosynthesis and the regulation of seed development.

Do the individual SL products of MAX1s and other SL biosynthesis enzymes act on specific plant traits? Our uncovering of the divergent nature of the potential pathways that could be regulating the individual MAX1s may indicate functional divergence, rather than simply redundancy or escape from parasitic weeds. CL and CLA are mobile and non-bioactive. Specific enzymes, like MAX1, may convert CLA to specific bioactive SLs near the site of action, which may induce tissue-specific responses. This is reminiscent of the gibberellin (GA) pathway where there are multiple different chemical forms, and major transported or stored forms, such as GA_12_, GA_20_ and GA_53_, are non-bioactive, and are converted to bioactive forms, such as GA_1_ and GA_4_, on-site [209,210]. Moreover, in bud outgrowth, GA_1_ and GA_4_ can promote bud elongation, whereas GA_3_ and GA_6_ seem to inhibit bud growth by deactivating GA_1_ and GA_4_ [211]. Other plant hormones also show diversity of chemical structure and activity. For example, isopentenyl-adenine and *trans*-zeatin cytokinins appear to have different functions [212].

Our predictions of new functions for SLs, and descriptions of the regulatory mechanisms for each of *MAX1* homologues, will facilitate the design of experiments, particularly using single mutants (in *MAX1s*, or TFs and miRNAs that are specific to the single *MAX1*). Also, analysis of the levels of different SLs that are produced and/or exuded by different genotypes needs to be performed during development processes or during responses to various treatments. Natural variation provided by the Bala rice cultivar could be a good place to begin those experimental approaches in combination with tissue-specific complementation.

## Figures and Tables

**Table 1:** Number of TFs found in promoter regions of *MAX1* homologues, categorised according to functions in plant development, growth and response to stresses.

	*Os* *01g0700900*	*Os* *01g0701400*	*Os* *01g0701500*	*Os* *02g0221900*	*Os* *06g0565100*
abiotic stresses					
iron status	1	0	1	0	4
nitrogen status	2	0	2	0	11
phosphorus status	0	1	0	0	2
arsenic	1	1	0	0	9
cadmium	2	0	1	0	8
chromium	0	0	1	1	0
cold	4	2	1	0	7
drought	2	1	3	1	20
salt	2	1	3	0	7
submergence	0	1	1	0	3
biotic stresses					
bacteria	1	1	1	0	11
viruses	1	0	2	0	0
fungi	1	0	2	0	17
insects	0	0	0	0	**11**
developmental processes					
plant height	**1**	0	0	0	0
shoot architecture	0	0	0	0	**6**
root development	1	1	2	1	6
flower development	1	1	3	2	2
seed development	0	0	0	**2**	0
seed dormancy	1	0	0	1	0
leaf senescence	1	0	1	0	0
secondary wall formation	0	0	**4**	0	0
wax synthesis	0	0	0	0	**1**
hormonal cross talk					
abscisic acid	0	1	0	0	1
ethylene	0	1	0	0	1
gibberelins	1	0	0	0	1

Bolded and underlined text indicates that this function was specific only to one *MAX1* homolog.

**Table 2:** MiRNAs that bind *MAX1* homologues categorised according to the functions they play in plant development, growth and response to stresses.

	*Os* *01g0700900*	*Os* *01g0701400*	*Os* *01g0701500*	*Os* *02g0221900*	*Os* *06g0565100*
abiotic stresses					
phosphorus status					*miR827*
zinc deficiency			*miR528-5p*		
cold			*miR528-5p*		
drought			*miR528-5p*		
heat	*miR2055*	*miR166d-5p*	*miR166b-5p* *miR528-5p* *miR5519*		*miR1848*
biotic stresses					
bacteria			*miR166b-5p*		
viruses	*miR1432-3p*	*miR166d-5p* *miR2097-3p*			
fungi			*miR2103*		
developmental processes					
flower development		*miR5514*	*miR528-5p*		
leaf senescence					*miR1848*
light signalling				*miR1430*	
wax synthesis					*miR1848*
hormonal cross talk					
abscisic acid			*miR528-5p*		
brassinosteroids					*miR1848*

**Table 3:** Function of genes co-expressed with *MAX1* homologues (MR < 100).

	*Os* *01g0700900*	*Os* *01g0701400*	*Os* *01g0701500*	*Os* *02g0221900*	*Os* *06g0565100*
abiotic stresses					
phosphorus status					*miR827*
zinc deficiency			*miR528-5p*		
cold			*miR528-5p*		
drought			*miR528-5p*		
heat	*miR2055*	*miR166d-5p*	*miR166b-5p* *miR528-5p* *miR5519*		*miR1848*
biotic stresses					
bacteria			*miR166b-5p*		
viruses	*miR1432-3p*	*miR166d-5p* *miR2097-3p*			
fungi			*miR2103*		
developmental processes					
flower development		*miR5514*	*miR528-5p*		
leaf senescence					*miR1848*
light signalling				*miR1430*	
wax synthesis					*miR1848*
hormonal cross talk					
abscisic acid			*miR528-5p*		
brassinosteroids					*miR1848*

## Supplementary Materials

The following are available online at https://www.mdpi.com/2073-4425/11/11/1348/s1, Supplementary File 1: Sequences and gene IDs. Figure S1: Alignment of rice MAX1 proteins, Table S1: TF motifs identified in the promoter region of rice MAX1 genes, Table S2: Common TF motifs identified in the promoter region of rice MAX1 genes, Table S3: TFs specific to Os01g0700900, Table S4: TFs specific to Os01g0701400, Table S5: TFs specific to Os01g0701500, Table S6: TFs specific to Os02g0221900, Table S7: TFs specific to Os06g0565100, Table S8: MiRNAs that bind rice MAX1 genes, Table S9: Expression profiles of rice MAX1 genes, Table S10: CGN of rice MAX1 genes. Table S11 GO for CGN of rice MAX1 genes, Table S12 Enrichment of GO for CGN of rice MAX1 genes.

## Abbreviations

4DO	4-deoxyorobanchol
5DS	5-deoxystrigol
ABA	abscisic acid
AM	arbuscular mycorrhizal
CCD	carotenoid cleavage dioxygenase
CGN	co-expression gene network
CL	carlactone
CLA	carlactonoic acid
D27	DWARF27
Edh1	Early headingdate1
EUI	ELONGATION OF UPPER MOST INTERNODE I
GAs	gibberelins
GEO	Gene Expression Omnibus
GO	Gene Ontology
LBO	LATERAL BRANCHING OXIDOREDUCTASE
LYP9	LiangYou-Pei 9
MAX1	MORE AXILLARY GROWTH1
MeCLA	methyl carlactonoate
Me-O-5-	methoxy-5-deoxystrigol
MR	Mutual Rank
N2Y6	Nei-2-You 6
nt	nucleotide
OsSWN1/2	Oryza sativa SECONDARY WALL NAC DOMAIN PROTEIN1/2
OsWS1	Oryza sativa wax synthase isoform 1
PRP	proline–rich protein
SL	strigolactones
TF	transcription factor
