# Supplementary material for: Diverse Roles of MAX1 Homologues in Rice
Source: Genes (Basel). 2020 Nov 13;11(11):1348. doi: 10.3390/genes11111348 (PMC7709044; doi:10.3390/genes11111348)
Supplement: Supplementary file 1 [file genes-11-01348-s001.zip › Table S3 TF specific to Os01g0700900.docx]

| **PlantPAN ID** | **Family** | **Position** | **Strand** | **Similar Score** | **Hit Sequence** | **TF ID or Motif name** |  |
| --- | --- | --- | --- | --- | --- | --- | --- |
| **TFmatrix**  **ID_0216** | C2H2 | 769 | **-** | 1 | gAGTGAa | Os01g0838600; Os01g0839100; Os03g0279700; Os03g0820300; Os03g0820400; Os05g0114400; Os05g0460900 |  |
| **Functions:**  Involved in response to drought (Shin 2016; Ahn, 2017), cold (da Maia), cadium (Ogawa 2009), nitrogen deficiency (Hsieh 2018), iron deficiency (Bashir 2014) | | | | | | |  |
| **TFmatrix**  **ID_0298** | Homeo-domain; HD-ZIP | 1024 | - | 1 | cAATCAtacc | Os02g0729700; Os03g0170600; Os03g0188900; Os08g0416000; Os08g0481400; Os09g0379600; Os09g0470500; Os10g0377300; Os10g0404900 |  |
|  |  | 1442 | - | 1 | cAATCAtgta |  |  |
| **Functions:**  Involved in response to salt (Hossain 2014), cold (Wang 2018), arsenic (Das 2018); involved in root development (Lavarenne) and seed dormancy/germination (Sugimoto 2009) | | | | | | |  |
| **TFmatrix**  **ID_0381** | NAC; NAM | 1690 | + | 0.98 | gtTACGTg | Os01g0104200; Os01g0816100; Os03g0327800; Os03g0815100; Os05g0421600; Os07g0225300; Os07g0566500; Os07g0683200; Os11g0126900; Os11g0184900; Os12g0123700 |  |
| **Functions:**  Involved in response to drought (Wang 2011, Shin 2016, Chung 2018), cold (Kitazumi 2018), cadium (Ogawa 2009), salt (Mito, 2011, Kim 2015), nitrogen deficiency (Yang 2017), viruses (Kikuchi, 2014), bacteria (Wang 2019), fungi (Tezuka 2019); involved in leaf senescence (Lee 2017, Yamatani 2013). | | | | | | |  |
| **TFmatrix**  **ID_0529** | MADS box; MIKC; M-type | 545 | + | 0.92 | agagCCATA tctgggagc | Os01g0201700; Os01g0883100; Os01g0886200; Os02g0104100; Os02g0170300; Os02g0682200; Os02g0731200; Os03g0122600; Os03g0752800; Os03g0753100; Os04g0580700; Os05g0203800; Os06g0162800; Os07g0108900; Os08g0494100; Os08g0531700; Os09g0507200; Os10g0536100; Os12g0207000; LOC_Os05g11380 |  |
| **Functions:**  Involved in response to cold (Kitazumi 2018); involved in flower development (Matsubara 2018); cross-talk with gibberellic acid (Chu 2019) | | | | | | |  |
