# Supplementary material for: Diverse Roles of MAX1 Homologues in Rice
Source: Genes (Basel). 2020 Nov 13;11(11):1348. doi: 10.3390/genes11111348 (PMC7709044; doi:10.3390/genes11111348)
Supplement: Supplementary file 1 [file genes-11-01348-s001.zip › Table S4 TF specific to Os01g0701400.docx]

| **PlantPAN ID** | **Family** | **Position** | **Strand** | **Similar Score** | **Hit Sequence** | **TF ID or Motif name** |
| --- | --- | --- | --- | --- | --- | --- |
| **TFmatrix**  **ID_0072** | AP2 | 2471 | **-** | 1 | gGCGGCaa | Os07g0617000; Os07g0674800; Os08g0537900; Os09g0286600; Os09g0287000; Os09g0369000; Os09g0434500; Os09g0457900; Os10g0390800; Os11g0168500; Os01g0313300; Os01g0797600; Os01g0868000; Os02g0521100; Os02g0546600; Os02g0654700; Os02g0655200; Os02g0764700; Os03g0183000; Os03g0183200; Os03g0183300; Os03g0191900; Os03g0341000; Os04g0398000; Os04g0429050; Os04g0546800; Os05g0361700; Os05g0437050; Os05g0497300; Os06g0194000; LOC_Os02g34270; |
| **Functions:**  Involved in root architecture (Kitomi 2018 Lee 2016), response to arsenic (Das, 2018 Singh 2017), cold (Zhang 2012), drought ((wang 2011) (Tula, 2013) (Mohanty, 2016) (Xu, 2017) (Chung 2018) (Ahn 2017)), phosphate deficiency (Oono, 2013), submergence (Mohanty, 2016), response to bacterial infection (Wang 2019), response to ethylene and abscisic treatment Sakamoto 2017 | | | | | | |
| **TFmatrix**  **ID_0261** | Dof; GATA | 123 | + | 1 | tAGATCt | Os03g0684000; Os02g0148500; Os03g0734900 |
|  |  | 1702 | + | 1 | tAGATCt |  |
|  |  | 1703 | - | 1 | aGATCTa |  |
| **Functions:**  Involved in response to cold (Nah, 2016) | | | | | | |
| **TFmatrix**  **ID_0503** | MADS box; MIKC; M-type | 262 | + | 0.87 | atcggacaaa  aggAGAAAtat | Os02g0682200; Os03g0122600; Os04g0580700; Os08g0494100; Os08g0531700; Os09g0507200; Os10g0536100 |
|  |  | 897 | - | 0.89 | actTTTCTa  atttctgtcagt |  |
|  |  | 1917 | - | 0.92 | tacTTTCTtt  tttccgttgca |  |
| **Functions:**  Involved in flowering (Das, 2018; Nguyen 2016; Kubo 2013 Fang, 2019 Tsuji 2011; Hori 2016) | | | | | | |
| **TF_motif_**  **seq_0187** | Motif seq only | 1632 | - | 0.74 | aattataaag  atagAGCAT | GLUTEBP2OS |
| **Functions:**  Regulate the transcription of genes encoding glutelin storage proteins (Croissant-Sych and Okita, 1996) | | | | | | |
| **TF_motif_**  **seq_0509** | Motif seq only | 1391 | - | 1 | aacaGGGAA | ANAERO5CONSENSUS |
| **Functions:**  One of 16 motifs found *in silico* in promoters of 13 anaerobicgenes involved in the fermentative pathway (anaerobic set 1) (Mohanty et al., 2005) | | | | | | |
