# Supplementary material for: Diverse Roles of MAX1 Homologues in Rice
Source: Genes (Basel). 2020 Nov 13;11(11):1348. doi: 10.3390/genes11111348 (PMC7709044; doi:10.3390/genes11111348)
Supplement: Supplementary file 1 [file genes-11-01348-s001.zip › Table S5 TF specific to Os01g0701500.docx]

| **PlantPAN ID** | **Family** | **Position** | **Strand** | **Similar Score** | **Hit Sequence** | **TF ID or Motif name** |
| --- | --- | --- | --- | --- | --- | --- |
| **TFmatrixID_0215** | C2H2 | 189 | **-** | 1 | gAGTGTg | Os01g0838600; Os03g0279700; Os05g0114400 |
| **Functions:**  Involved in response to iron deficiency (Bashir, 2014), (Le 2016); nitrogen deficiency (Kan, 2015; Hsieh 2018; Yang 2017). | | | | | | |
| **TFmatrixID_0219** | C2H2 | 189 | - | 1 | gAGTGTg | Os03g0437100; Os03g0437200; Os12g0583700 |
| **Functions:**  Involved in response to cold (Fujino 2010); drought (Cui, 2018; Gao, 2019; Ahn, 2017); salt (Kim, 2015); cadium (Ogawa, 2009). | | | | | | |
| **TFmatrixID_0386**  **TFmatrixID_0388**  **TFmatrixID_0389**  **TFmatrixID_0390**  **TFmatrixID_0395** | NAC; NAM | 2319 | + | 0.96 | ttGCGTGg | Os01g0104500; Os01g0393100; Os02g0579000; Os03g0327100; Os03g0624600; Os04g0460600; Os06g0344900; Os07g0684800; Os08g0511200; Os09g0497900; Os11g0127600; Os12g0123800 |
|  |  | 2318 | + | 0.99 | tttGCGTGg |  |
|  |  | 2317 | + | 0.99 | ttttGCGTGg |  |
|  |  | 2317 | + | 0.99 | ttttGCGTGg |  |
|  |  | 2319 | + | 0.99 | ttGCGTGgag |  |
| **Functions:**  Involved in response to viruses (Kikuchi, S. 2014); fungi (Tezuka 2019); drought (Shin, 2016; Ahn, 2017); flower development (Kim, 2017). | | | | | | |
| **TFmatrixID_0392** | NAC; NAM | 2319 | + | 1 | tTGCGTggag | Os02g0252200; Os02g0643600; Os06g0104200; Os06g0131700; Os06g0530400; Os08g0103900; Os08g0115800; Os10g0532000; LOC_Os03g03540; |
| **Functions:**  Involved in response to viruses Kikuchi, S. (2014); secondary cell wall formation (Hirano, 2013; Hirano 2013; Yoshida 2013; Zhng 2011); root development (Kitomi, 2018) | | | | | | |
| **TFmatrixID_0499** | MADS box; MIKC; M-type | 1665 | - | 0.89 | cttatttattt  aTTTTTtttg | Os01g0201700; Os01g0883100; Os01g0886200; Os02g0104100; Os02g0170300; Os02g0579600; Os02g0682200; Os02g0731200; Os03g0122600; Os03g0752800; Os03g0753100; Os04g0461300; Os04g0580700; Os05g0203800; Os06g0108500; Os06g0162800; Os06g0667200; Os07g0108900; Os08g0431900; Os08g0494100; Os08g0531700; Os08g0531900; Os09g0507200; Os10g0536100; Os12g0207000 |
| **Functions:**  Involved in response to salt (Tuteja, 2015); flower development (Yasui 2017; Nguyen 2016; Ohnishi 2011; Sato 2012; Kuwano 2011; Ke 2018) | | | | | | |
| **TFmatrixID_0524**  **TFmatrixID_0547** | Myb/  SANT | 2042 | + | 0.95 | atcGCTGAgat | Os04g0348300 |
|  |  | 2042 | + | 0.95 |  |  |
| **Functions:**  Ubiquitous expression in flower buds, roots after flowering [www.dna.affrc.go.jp/PLACE/] | | | | | | |
| **TFmatrixID_0562** | EIN3 | 1027 | + | 0.98 | ATGTAtct | Os03g0324200; Os03g0324300; Os07g0685700; Os08g0508700 |
| **Functions:**  Involved in response to fungi (Tezuka 2019); drought (Shin, 2016); chromium (Huang 2014); submergence Xiong 2012); leaf senescence (Piao 2015; Lee 2015). | | | | | | |
| **TF_motif_seq_0175** |  | 761 | - | 0.67 | tgaacttattagaGATAT | GLUTEBOX1OSGT2 |
| **TF_motif_seq_0175** |  | 938 | - | 0.61 | ATATCttcacatacataa |  |
| **Functions:**  Box 1 of rice (O.s.) glutelin Gt2 gene family promoter regions; nuclear factor binding site [www.dna.affrc.go.jp/PLACE/] | | | | | | |
| **TF_motif_seq_0470** | HD-ZIP | 993 | + | 1 | CAATAattg | Os08g0416000; Os09g0470500; Os09g0528200 |
|  |  | 993 | - | 1 | caatAATTG |  |
|  |  | 1509 | + | 1 | CAATTattg |  |
|  |  | 1509 | - | 1 | caatTATTG |  |
| **Functions:**  Involved in response to bacteria (King 2019); salt (Hossain, 2014) | | | | | | |
