# Supplementary material for: Diverse Roles of MAX1 Homologues in Rice
Source: Genes (Basel). 2020 Nov 13;11(11):1348. doi: 10.3390/genes11111348 (PMC7709044; doi:10.3390/genes11111348)
Supplement: Supplementary file 1 [file genes-11-01348-s001.zip › Table S6 TF specific to Os02g0221900.docx]

| **PlantPAN ID** | **Family** | **Position** | **Strand** | **Similar Score** | **Hit Sequence** | **TF ID or Motif name** |
| --- | --- | --- | --- | --- | --- | --- |
| **TFmatrixID_0304** | LOB | 1380 | **+** | 0.99 | aataTGCGGg | Os01g0169400 ;Os03g0659700; Os05g0346800; Os11g0106900; Os12g0106200 |
| **Functions:**  Involved in root development (Kitomi 2012; Kortz 2019) | | | | | | |
| **TFmatrixID_0421** | TCP | 653 | - | 0.97 | tGGTGCtttc | Os01g0763200; Os03g0706500; Os03g0785800; Os05g0513100; Os07g0152000; Os12g0616400; LOC_Os07g04510; LOC_Os12g02090; |
|  |  | 914 | + | 0.97 | cataGCACCa |  |
|  |  | 960 | - | 0.97 | tGGTGCtaat |  |
|  |  | 1983 | + | 0.97 | agcaGCACCa |  |
|  |  | 1995 | + | 0.97 | ccagGCACCa |  |
| **Functions:**  Involved in response to cold (Yang, 2013); flower development (Gupta, 2017), axillary bud outgrowth (Minakuchi), cell division that is contolled by SL and CK (Hu, 2013);  OsTCP5 is involved in the control of cell division by SL and CK in the mesocotyl | | | | | | |
| **TFmatrixID_0509** | B3 | 2047  2048 | -  + | 0.96  0.97 | gaGCATGca | Os01g0911700 |
| **Functions:**  Involved in response to chromium (Huang 2014), seed development Miyoshi 2002; | | | | | | |
| **TFmatrixID_0551** | Myb  /SANT; MYB | 2389 | + | 0.95 | gttGGTTG | Os01g0812000 |
| **Functions:**  Involved in response to drought (Jin 2018); flower development Fu 2014; | | | | | | |
| **TFmatrixID_0579** | Myb/  SANT | 828 | + | 0.84 | gtgagt  TAACAtgca | Os04g0348300 |
| **Functions:**  Unknown function | | | | | | |
| **TF_motif_seq_0189** | Motif sequence only | 2063 | + | 0.77 | TCCATgga  ggcaa | RYREPEAT4 |
| **Functions:**  RY repeat motif; "Sph element"; seed expression (http://plantpan.itps.ncku.edu.tw/) | | | | | | |
| **TF_motif_seq_0124** | Motif sequence only | 911 | + | 0.71 | AAGCA  tagcaccac | GLUTEBP1OS |
| **Functions:**  Glutelin BP-1; Binding site in the promoter region of glutelinGt3 gene family of nuclear factor (PB-1); PB-1 is observed only in nuclear extract of developing seeds (http://plantpan.itps.ncku.edu.tw/) | | | | | | |
| **TF_motif_seq_0189** | Motif sequence only | 2308 | - | 0.74 | gatcttcag  gtaatGATGC | ABFOS |
| **Functions:**  ABF (as-1-like box binding factor) binding site; as-1-like (ASL)box is found at -98 to -79 of RTBV (rice tungro bacilliform virus) promoter; ASL box is required for phloem-specific gene expression of Rice Tungro Bacilliform Virus (RTBV) (http://plantpan.itps.ncku.edu.tw/) | | | | | | |
