# Supplementary material for: Diverse Roles of MAX1 Homologues in Rice
Source: Genes (Basel). 2020 Nov 13;11(11):1348. doi: 10.3390/genes11111348 (PMC7709044; doi:10.3390/genes11111348)
Supplement: Supplementary file 1 [file genes-11-01348-s001.zip › Table S7 TF specific to Os06g0565100.docx]

| **PlantPAN ID** | **Family** | **Position** | **Strand** | **Similar Score** | **Hit Sequence** | **TF ID or Motif name** |
| --- | --- | --- | --- | --- | --- | --- |
| **TFmatrixID_0053** | AP2 | 674  2053 | **+**  **-** | 1  1 | CGCCGcca  tggCGGCG | Os06g0691100; Os07g0669500; Os07g0674800; Os08g0537900; Os09g0287000; Os09g0457900; Os10g0390800; Os11g0168500; Os01g0224100; Os01g0752500; Os01g0797600; Os01g0868000; Os02g0189500; Os02g0520000; Os02g0521100; Os02g0546600; Os02g0594300; Os02g0654700; Os02g0655200; Os03g0150200; Os04g0257500; Os04g0398000; Os04g0399800; Os04g0546800; Os04g0610400; Os04g0669200; Os05g0361700; Os05g0437050; Os05g0497300; LOC_Os10g30840; LOC_Os02g06330; |
| **Functions:**  Involved in response to bacteria Xu 2013; fungi Wang 2019; drought Mohanty 2016, Xu 2017, Wang 2011; phosphorus deficiency Oono 2013; flower development Fujishiro 2018; root development Kitomi 2012; Kortz 2019; | | | | | | |
| **TFmatrixID_0054** | AP2 | 73  76  220  671  692  2056  2059  2062 | +  +  +  +  +  -  -  - | 1  1  1  1  1  1  1  1 | cgCCGCCg  cgCCGCCg  cgCCGCCg  cgCCGCCg  cgCCGCCg  cGGCGGcg  cGGCGGcg  cGGCGGcg | Os07g0410300; Os09g0571700; Os09g0572000; Os10g0562900; Os02g0654700; Os03g0150200; Os03g0860100; Os04g0257500; Os04g0546800; Os05g0448675; Os05g0564700; LOC_Os08g44960; LOC_Os10g30840; |
| **Functions:**  Involved in response to bacteria Xu 2011; fungi Tezuka 2019; cadmium Sun 2019; nitrogen deficiency Kan 2015 Sinha 2018; | | | | | | |
| **TFmatrixID_0055** | AP2 | 75  219  673  2052  2055  2058  2061 | +  +  +  -  -  -  - | 1  1  0.99  0.99  1  1  1 | cCGCCGccgt  cCGCCGccgc  cCGCCGccac  gtggCGGCGg  gcggCGGCGg  gcggCGGCGg  gcggCGGCGg | Os06g0604000; Os02g0202000; Os02g0797100; Os04g0655700; Os06g0181700 |
| **Functions:**  Involved in response to drought and salt Wang 2012; submergence Lakshmanan 2014; wax synthesis Wang 2012; response to ethylene and abscisic acid | | | | | | |
| **TFmatrixID_0061** | AP2 | 73  76  220  671  674  692  2053  2056  2059  2062 | +  +  +  +  +  +  -  -  -  - | 0.98  0.98  0.98  0.98  1  0.98  1  0.98  0.98  0.98 | CGCCGccg  CGCCGccg  CGCCGccg  CGCCGccg  CGCCGcca  CGCCGccg  tggCGGCG  cggCGGCG  cggCGGCG  cggCGGCG | Os06g0691100; Os08g0537900; Os09g0287000; Os10g0390800; Os11g0168500; Os01g0224100; Os01g0657400; Os01g0797600; Os01g0868000; Os02g0189500; Os02g0520000; Os02g0546600; Os02g0594300; Os02g0654700; Os03g0150200; Os03g0815800; Os03g0860100; Os04g0399800; Os04g0610400; Os04g0669200; Os05g0361700; Os05g0437050; Os05g0497300; Os06g0160500; LOC_Os10g30840; LOC_Os02g06330; |
| **Functions:**  Involved in response to bacteria Xu 2011, fungi Wang 2019; insects Wang, 2012; drought Wang 2011; Mohanty 2017 Chung, 2018 Xu 2017; cadmium Zhang 2012; Sun 2019; iron deficiency Finatto 2015; control of ABA and GAs biosynthesis | | | | | | |
| **TFmatrixID_0066** | AP2; ERF | 216  842 | +  - | 1  1 | gCACCGccg  gCACCGccg | Os08g0565200; Os01g0165000; Os03g0174400; Os03g0191900; Os05g0346200 |
| **Functions:**  Involved in response to fungi Yi 2013; cold Zhao 2015; drought Jin 2018; salt Wu 2019 | | | | | | |
| **TFmatrixID_0068** | AP2 | 40  258  288  377  445  533  592  2114  2114  2179  2179 | +  -  -  -  -  -  -  +  -  +  - | 0.99  0.99  0.99  0.99  0.99  0.99  0.99  0.97  0.97  0.99  0.99 | agCCGGCc  gGCCGGca  gGCCGGca  gGCCGGca  gGCCGGca  gGCCGGca  gGCCGGca  cgCCGGCg  cGCCGGcg  ggCCGGCc  gGCCGGcc | Os07g0410300; Os09g0571700; Os09g0572000; Os10g0562900; Os01g0752500; Os02g0654700; Os03g0150200; Os03g0860100; Os04g0257500; Os04g0546800; Os05g0448675; Os05g0564700; LOC_Os10g30840; LOC_Os08g44960; |
| **Functions:**  Involved in response to fungi Tezuka 2019; drought Wang 2011; cadmium Sun 2019; nitrogen deficiency Kan 2015; Sinha,2018; Yang, 2017; | | | | | | |
| **TFmatrixID_0069** | AP2 | 74  218  672  2053  2056  2059  2062 | +  +  +  -  -  -  - | 0.99  0.99  1  1  0.99  0.99  0.99 | \| gcCGCCGcc  acCGCCGcc  gcCGCCGcc  tggCGGCGg  cggCGGCG  cggCGGCG  cggCGGCG \| \| --- \| | Os06g0222400; Os07g0617000; Os07g0674800; Os08g0537900; Os09g0287000; Os09g0369000; Os09g0434500; Os09g0457900; Os10g0390800; Os10g0562900; Os11g0168500; Os01g0313300; Os01g0752500; Os01g0797600; Os01g0868000; Os02g0521100; Os02g0546600; Os02g0654700; Os02g0655200; Os02g0752800; Os02g0764700; Os03g0150200; Os03g0183000; Os03g0183300; Os03g0191900; Os03g0341000; Os04g0257500; Os04g0398000; Os04g0429050; Os04g0546800; Os04g0547500; Os04g0547600; Os04g0610400; Os05g0361700; Os05g0437050; Os05g0497300; Os05g0564700; LOC_Os10g30840; LOC_Os02g34270; |
| **Functions:**  Involved in response to bacteria (Brusamarello-Santos 2012); fungi Lilly 2019 Wang 2019; drought Chung 2018, Wang 2011; arsenic Das 2018 | | | | | | |
| **TFmatrixID_0071** | AP2 | 674  2053 | +  - | 1  1 | cgCCGCCa  tGGCGGcg | Os10g0562900; Os01g0752500; Os01g0868000; Os02g0546600; Os02g0654700; Os02g0655200; Os03g0150200; Os04g0257500; Os04g0546800; Os04g0547500; Os04g0547600; Os05g0437050; LOC_Os10g30840 |
| **Functions:**  Involved in response to bacteria Xu 2011, drought Xu 2017 Wang 2011; arsenic Singh 2017; nitrogen deficiency Sinha 2018; Yang,2017; | | | | | | |
| **TFmatrixID_0077** | AP2 | 76  220  674  692  2051  2219 | +  +  +  +  -  + | 1  1  1  1  1  0.99 | cGCCGCcgta  cGCCGCcgct  cGCCGCcacc  cGCCGCcgct  agtgGCGGCg  cGCCGCccat | Os06g0691100; Os08g0537900; Os10g0390800; Os11g0168500; Os01g0797600; Os02g0189500; Os02g0546600; Os02g0594300; Os04g0399800; Os04g0610400; Os04g0669200; Os05g0497300; LOC_Os02g06330 |
| **Functions:**  Involved in response to fungi He 2016; cold Zhang 2012; drought Mohanty 2016, Wang, 2011 Xu 2017; submergence Kottapalli 2007; arsenic Singh 2017 | | | | | | |
| **TFmatrixID_0081** | AP2 | 257  257  287  287  376  376  444  532  532  591  591  2113  2113  2178 | +  -  +  -  +  -  +  +  -  +  -  +  -  + | 0.99  0.98  0.99  0.98  0.99  0.98  0.99  0.99  0.98  0.99  0.98  0.99  0.99  0.98 | cgGCCGGcag  cggCCGGCag  cgGCCGGcag  cggCCGGCag  cgGCCGGcag  cggCCGGCag  tgGCCGGcag  cgGCCGGcag  cggCCGGCag  cgGCCGGcag  cggCCGGCag  tcGCCGGcgt  tcgCCGGCgt  ggGCCGGccg | Os09g0571700; Os10g0390800; Os10g0562900; Os01g0752500; Os01g0868000; Os02g0654700; Os02g0655200; Os03g0150200; Os03g0183000; Os03g0860100; Os04g0257500; Os04g0429050; Os04g0546800; Os04g0547500; Os04g0547600; Os05g0437050; Os05g0564700; LOC_Os10g30840; |
| **Functions:**  Involved in response to bacteria Xu 2011; fungi Tezuka 2019; drought Wang 2011, Shin,2016; cadmium Sun 2019; iron deficiency Finatto 2015; nitrogen deficiency Sinha 2018; Yang, 2017; phosphorus deficiency Oono 2013; | | | | | | |
| **TFmatrixID_0083** | AP2 | 219  673  2054  2057  2060  2063 | +  +  -  -  -  - | 1  1  1  1  1  1 | ccGCCGCc  ccGCCGCc  gGCGGCgg  gGCGGCgg  gGCGGCgg  gGCGGCgg | Os09g0434500; Os11g0168500; Os01g0224100; Os01g0657400 |
| **Functions:**  Involved in response to insects Wang 2012; drought Mohanty 2016, Chung, 2018; | | | | | | |
| **TFmatrixID_0087** | AP2 | 75  219  673  2054  2057  2060  2063 | +  +  +  -  -  -  - | 0.99  0.99  0.99  0.99  0.99  0.99  0.99 | cCGCCGcc  cCGCCGcc  cCGCCGcc  ggCGGCGg  ggCGGCGg  ggCGGCGg  ggCGGCGg | Os08g0408500; Os09g0369000; Os11g0168500; Os01g0224100; Os01g0657400; Os03g0183200; Os03g0191900; Os04g0429050 |
| **Functions:**  Involved in response to fungi Wang 2019; insects Wang 2012; cold Zhang 2012; drought Chung 2018 Wang 2011, Shin 2016; salt Tula 2013; | | | | | | |
| **TFmatrixID_0092** | AP2 | 85  673  2053  2065 | +  +  -  - | 1  1  1  0.99 | aggCCGCCa  ccgCCGCCa  tGGCGGcgg  cGGCGGctg | Os10g0562900; Os02g0546600; Os02g0654700; Os02g0655200; Os04g0546800; Os04g0547500; Os04g0547600 |
| **Functions:**  Involved in response to drought Wang 2011, Xu 2017; arsenic Singh 2017; nitrogen deficiency Sinha 2018; | | | | | | |
| **TFmatrixID_0100** | AP2 | 75  219  673  2052  2055  2058  2061  2218 | +  +  +  -  -  -  -  + | 1  1  1  1  1  1  1  0.99 | cCGCCGccgt  cCGCCGccgc  cCGCCGccac  gtggCGGCGg  gcggCGGCGg  gcggCGGCGg  gcggCGGCGg  tCGCCGccca | Os07g0617000; Os07g0674800; Os08g0537900; Os09g0434500; Os09g0457900; Os10g0562900; Os11g0168500; Os01g0313300; Os01g0752500; Os01g0797600; Os01g0868000; Os02g0521100; Os02g0546600; Os02g0654700; Os02g0655200; Os02g0764700; Os03g0150200; Os03g0183000; Os03g0183300; Os03g0341000; Os04g0257500; Os04g0398000; Os04g0429050; Os04g0546800; Os05g0437050; Os05g0564700; LOC_Os02g34270; LOC_Os10g30840 |
| **Functions:**  Involved in response fungi Wang 2019, Tezuka 2019; drought Wang 2011 Mohanty 2016; arsenic Das 2018; nitrogen deficiency Sinha 2018; | | | | | | |
| **TFmatrixID_0101** | AP2 | 72  75  219  670  673  691  2052  2055  2058  2061 | +  +  +  +  +  +  -  -  -  - | 1  1  1  1  1  1  1  1  1  1 | tcGCCGCcgc  ccGCCGCcgt  ccGCCGCcgc  tcGCCGCcgc  ccGCCGCcac  tcGCCGCcgc  gtgGCGGCgg  gcgGCGGCgg  gcgGCGGCgg  gcgGCGGCgg | Os09g0457900; Os09g0571700; Os10g0562900; Os11g0168500; Os01g0657400; Os01g0752500; Os01g0797600; Os02g0546600; Os02g0654700; Os02g0655200; Os02g0764700; Os03g0150200; Os03g0860100; Os04g0257500; Os04g0399800; Os04g0429050; Os04g0546800; Os04g0547500; Os04g0547600; Os05g0497300; Os05g0564700; LOC_Os08g44960; LOC_Os10g30840; |
|  |  |  |  |  |  |  |
| **Functions:**  Involved in response to bacteria Xu 2011; fungi Tezuka 2019; cold Zhang 2012; drought Chung; arsenic Singh; nitrogen deficiency Sinha 2018, Yang 2017; root development Kitomi 2018, | | | | | | |
| **TFmatrixID_0106** | AP2 | 40  258  258  288  288  377  377  445  445  533  533  592  592  2114  2114  2179  2179 | +  +  -  +  -  +  -  +  -  +  -  +  -  +  -  +  - | 0.94  0.97  0.94  0.97  0.94  0.97  0.94  0.97  0.94  0.97  0.94  0.97  0.94  0.97  0.97  0.94  0.94 | aGCCGGcc  gGCCGGca  ggCCGGCa  gGCCGGca  ggCCGGCa  gGCCGGca  ggCCGGCa  gGCCGGca  ggCCGGCa  gGCCGGca  ggCCGGCa  gGCCGGca  ggCCGGCa  cGCCGGcg  cgCCGGCg  gGCCGGcc  ggCCGGCc | Os08g0537900; Os09g0571700; Os10g0562900; Os01g0224100; Os01g0657400; Os01g0752500; Os01g0797600; Os01g0868000; Os02g0521100; Os02g0546600; Os02g0654700; Os02g0655200; Os03g0150200; Os04g0257500; Os04g0546800; Os04g0547500; Os04g0547600; Os05g0437050; Os05g0497300; LOC_Os10g30840; |
| **Functions:**  Involved in response to bacteria Xu 2011; fungi Tezuka 2019; insects Wang 2012; drought Mohanty 2016, Wang 2011, Xu 2017, Chung 2018; nitrogen deficiency Sinha 2018 Yang 2017, | | | | | | |
| **TFmatrixID_0107** | AP2 | 75  219  673  2053  2056  2059  2062 | +  +  +  -  -  -  - | 1  1  1  1  1  1  1 | cCGCCGccg  cCGCCGccg  cCGCCGcca  tggCGGCGg  cggCGGCGg  cggCGGCGg  cggCGGCGg | Os08g0408500; Os09g0369000; Os10g0390800; Os11g0168500; Os01g0224100; Os01g0657400; Os01g0868000; Os03g0191900; Os03g0815800; Os05g0437050; Os06g0160500 |
| **Functions:**  Involved in response to fungi Yi 2013; insects Wang 2012; cold Suzuki 2015; drought Wang 2011, Chung 2018, Xu 2017; salt Tula 2013; root development Jung 2017; | | | | | | |
| **TFmatrixID_0110** | AP2 | 73  76  220  671  674  692  2053  2056  2059  2062 | +  +  +  +  +  +  -  -  -  - | 1  1  1  1  1  1  1  1  1  1 | CGCCGccg  CGCCGccg  CGCCGccg  CGCCGccg  CGCCGcca  CGCCGccg  tggCGGCG  cggCGGCG  cggCGGCG  cggCGGCG | Os10g0562900; Os01g0797600; Os02g0189500; Os02g0520000; Os04g0399800; Os04g0547500; Os04g0547600; Os04g0669200; Os05g0497200; Os05g0497300 |
| **Functions:**  Involved in response to cold Zhang 2012; drought Xu 2017; nitrogen deficiency Sinha 2018; root development Kitomi, 2018 | | | | | | |
| **TFmatrixID_0113** | AP2; ERF | 74  218  672  2053  2056  2059  2062 | +  +  +  -  -  -  - | 1  1  1  1  1  1  1 | gccGCCGCcg  accGCCGCcg  gccGCCGCca  tgGCGGCggc  cgGCGGCggc  cgGCGGCggc  cgGCGGCggc | Os07g0617000; Os07g0674800; Os08g0474000; Os08g0537900; Os09g0369000; Os09g0434500; Os09g0457900; Os09g0571700; Os10g0390800; Os10g0562900; Os11g0168500; Os01g0224100; Os01g0313300; Os01g0657400; Os01g0752500; Os01g0797600; Os01g0868000; Os02g0521100; Os02g0546600; Os02g0654700; Os02g0655200; Os02g0764700; Os03g0150200; Os03g0183200; Os03g0191900; Os03g0341000; Os04g0257500; Os04g0398000; Os04g0429050; Os04g0546800; Os04g0547500; Os04g0547600; Os04g0610400; Os05g0351200; Os05g0437050; Os05g0497300; Os06g0194000; LOC_Os02g34270; LOC_Os10g30840; LOC_Os08g44960 |
| **Functions:**  Involved in response fungi Wang 2019, Tezuka, 2019; drought Wang; cadmium Ogawa 2009; salt Tula 2013; arsenic Das 2018; | | | | | | |
| **TFmatrixID_0122** | AP2 | 76  220  671  674  692  2053  2056  2059  2062 | +  +  +  +  +  -  -  -  - | 0.99  0.99  0.99  1  0.99  1  0.99  0.99  0.99 | cGCCGCcg  cGCCGCcg  cGCCGCcg  cGCCGCca  cGCCGCcg  tgGCGGCg  cgGCGGCg  cgGCGGCg  cgGCGGCg | Os07g0617000; Os09g0369000; Os09g0434500; Os09g0457900; Os10g0390800; Os01g0752500; Os01g0797600; Os02g0189500; Os02g0520000; Os02g0654700; Os03g0150200; Os03g0183200; Os03g0183300; Os03g0860100; Os04g0257500; Os05g0497300; Os06g0194000; LOC_Os10g30840; |
| **Functions:**  Involved in response to bacteria Xu 2011, fungi Wang 2019; submergence Mohanty 2016, salt Tula 2013; arsenic Das 2018; nitrogen deficiency Yang 2017; root development Kitomi 2018 | | | | | | |
| **TFmatrixID_0303** | LOB | 764 | + | 0.99 | cacCCGGGgt | Os03g0659700; Os05g0346800; Os11g0106900; Os12g0106200 |
| **Functions:**  Involved in root development Kitomi, 2012, Kortz, 2019 | | | | | | |
| **TFmatrixID_0426** | TCP | 937 | - | 0.99 | GTGGGtccct | Os01g0924400; Os02g0747400; Os04g0194600; Os04g0526000; Os06g0226700; Os09g0521300; Os12g0173300 |
| **Functions:**  Involved in response to insects Yuexiong 2019;  shoot architecture Lu 2013 | | | | | | |
| **TFmatrixID_0430** | TCP | 937 | - | 0.95 | GTGGGtcc | Os01g0763200; Os03g0706500; Os03g0785800; Os05g0513100; Os07g0152000; LOC_Os12g02090; LOC_Os07g04510; |
| **Functions:**  Involved in response to salt Wu 2019; flower development Gupta 2017; shoot architecture Lu 2013 | | | | | | |
| **TFmatrixID_0433 TFmatrixID_0435**  **TFmatrixID_0438**  **TFmatrixID_0439** | TCP | 937 | - | 0.98  0.96  0.99  0.99 | GTGGGtccct  GTGGGtcc  GTGGGtccc  GTGGGtcc | Os01g0924400; Os02g0747400; Os04g0194600; Os04g0526000; Os06g0226700; Os09g0521300; Os12g0173300 |
| **Functions:**  Involved in response to insects Yuexiong 2019; shoot architecture Lu 2013; | | | | | | |
| **TFmatrixID_0487** | AP2; ERF | 845  867  897 | +  +  + | 0.97  0.97  0.97 | CGGTGcatcc  CGGTGcacct  CGGTGcatcc | Os06g0222400; Os08g0537900; Os08g0565200; Os09g0369000; Os09g0457900; Os10g0371100; Os11g0168500; Os01g0868000; Os02g0546600; Os02g0638650; Os02g0656600; Os02g0657000; Os02g0752800; Os02g0764700; Os03g0174400; Os03g0191900; Os04g0529100; Os04g0549700; Os04g0549800; Os04g0550200; Os05g0346200; Os05g0351200; Os05g0437050 |
| **Functions:**  Involved in response to fungi Lilly 2019; cold Wang 2018; Kitazumi 2018; drought Chung 2018; Mohanty, 2016, Xu 2017; salt Tula 2013 Jin 2013; arsenic Singh 2017; | | | | | | |
| **TFmatrixID_0512** | AP2 | 674  2053 | +  - | 1  1 | cGCCGCca  tgGCGGCg | Os07g0410300; Os07g0410700; Os09g0571700; Os09g0572000; Os01g0752500; Os02g0654700; Os03g0150200; Os03g0860100; Os04g0257500; Os04g0546800; Os05g0448675; Os05g0564700; LOC_Os10g30840; LOC_Os08g44960; |
| **Functions:**  Involved in response to bacteria Xu 2011; fungi Tezuka 2019; insects Wang, 2012; drought Ahn 2017; cadmium Sun 2019; iron deficiency Finatto 2015; nitrogen Kan 2015; Hsieh, 2018; | | | | | | |
