# Supplementary material for: Diverse Roles of MAX1 Homologues in Rice
Source: Genes (Basel). 2020 Nov 13;11(11):1348. doi: 10.3390/genes11111348 (PMC7709044; doi:10.3390/genes11111348)
Supplement: Supplementary file 1 [file genes-11-01348-s001.zip › Table S8 MiRNAs that bind rice MAX1 genes.docx]

| ***Os01g0700900*** | | | | | | |
| --- | --- | --- | --- | --- | --- | --- |
| miRNA_Acc. | Expectation | UPE$ | Target_start | Target_end | miRNA-target alignment | Inhibition |
| osa-miR5144-5p | 3.0 | -1.0 | 906 | 926 | UUCUUGUGCUGCUGAAGAGAC  : : :: :::::::.::::.:  GGCGCUGCAGCAGCGCAAGGA | Cleavage |
| osa-miR419 | 4 | -1.0 | 662 | 682 | UGAUGAAUGCUGACGAUGUUG  ..:::: ::.: .:::::::  AGGCAUCAUCGGAGUUCAUCA | Cleavage |
| osa-miR2055 | 4.5 | -1.0 | 1271 | 1291 | UUUCCUUGGGAAGGUGGUUUC  :: :.: :::::: ::::.::  GAUAUCCCCUUCCAAAGGGAA | Cleavage |
| osa-miR1432-3p | 5 | -1.0 | 1292 | 1312 | CAGGUGUCAUCUCCCCUGAAC  : .: : .::::.::::::  CAUGGGUGUGGAUGGCACCUG | Cleavage |
| osa-miR160a-3p | 5 | -1.0 | 78 | 98 | GCGUGCAAGGAGCCAAGCAUG  .:: : :::: ::::.:::  GGUGGUAGGCUACUUGUACGA | Translation |
| osa-miR160b-3p | 5 | -1.0 | 78 | 98 | GCGUGCAAGGAGCCAAGCAUG  .:: : :::: ::::.:::  GGUGGUAGGCUACUUGUACGA | Translation |
| osa-miR1853-3p | 5 | -1.0 | 1556 | 1577 | UAAUUGGGGAUGUUCGGUUGCU  :.: .::: :.::.::::::  UGUACUCGAUCGUCUCCAAUUU | Cleavage |
| osa-miR1862a | 5 | -1.0 | 585 | 607 | ACGAGGUUGGUUUAUUUUGGGACG  : : .:.::: ::.....::::::  CCUGUCGAAA-AAGUUGGCCUCGU | Cleavage |
| osa-miR1862b | 5 | -1.0 | 585 | 607 | ACGAGGUUGGUUUAUUUUGGGACG  : : .:.::: ::.....::::::  CCUGUCGAAA-AAGUUGGCCUCGU | Cleavage |
| osa-miR1862c | 5 | -1.0 | 585 | 607 | ACGAGGUUGGUUUAUUUUGGGACG  : : .:.::: ::.....::::::  CCUGUCGAAA-AAGUUGGCCUCGU | Cleavage |
| osa-miR440 | 5 | -1.0 | 1592 | 1615 | AGUGUCUCCUGAUGAUCGGGACAA  :.: :.:::::: :::.::.  AGCUUCAGGUCAUCAAGAGGCAUA | Cleavage |
| osa-miR5490 | 5 | -1.0 | 1313 | 1333 | UUGGAUUUUUAUUUAGGACGG  .::::::.. ::..:::: :  GUGUCCUAGGAAAGGAUCCCA | Translation |
| ***Os01g0701400*** | | | | | | |
| osa-miR419 | 4.0 | -1.0 | 659 | 679 | UGAUGAAUGCUGACGAUGUUG  ..:::: ::.: .:::::::  AGGCAUCAUCGGAGUUCAUCA | Cleavage |
| osa-miR160a-3p | 4.5 | -1.0 | 78 | 98 | GCGUGCAAGGAGCCAAGCAUG  ::.: :::: ::::.:::  GCUGUUGGGCUACUUGUACGA | Translation |
| osa-miR160b-3p | 4.5 | -1.0 | 78 | 98 | GCGUGCAAGGAGCCAAGCAUG  ::.: :::: ::::.:::  GCUGUUGGGCUACUUGUACGA | Translation |
| osa-miR440 | 4.5 | -1.0 | 1595 | 1618 | AGUGUCUCCUGAUGAUCGGGACAA  :.: :.:::::: :::.:::  AGCUUCGGGUCAUCAAGAGGCACA | Cleavage |
| osa-miR1437b-3p | 5 | -1.0 | 660 | 683 | GUGCUGGCGAGCUCCGGUGCCGCA  :: ::.:::::.:: .::: :.  GGCAUCAUCGGAGUUCAUCAGGAU | Cleavage |
| osa-miR160d-3p | 5 | -1.0 | 78 | 98 | GCGUGCGAGGAGCCAAGCAUG  ::.: :::: ::.:.:::  GCUGUUGGGCUACUUGUACGA | Translation |
| osa-miR166d-5p | 5 | -1.0 | 383 | 403 | GGAAUGUUGUCUGGCUCGAGG  .::::: ..:::..:.:: :  GUUCGAGGUGGACGGCGUUGC | Cleavage |
| osa-miR2097-3p | 5 | -1.0 | 1384 | 1405 | UUCUCUUCUUCGUGUCGCAUUU  :: :::: . :.:::::::.:  AACGGCGAGGAGGAGAAGAGGA | Cleavage |
| osa-miR5514 | 5 | -1.0 | 52 | 72 | UCCCAGAGCUUUGGCCGUCGC  .:..::::: .::: ::::.  UUGGUGGCCAUGGCUGUGGGG | Translation |
| ***Os01g0701500*** | | | | | | |
| osa-miR419 | 3.0 | -1.0 | 656 | 676 | UGAUGAAUGCUGACGAUGUUG  ..:::::::.: .:::::::  AGGCAUCGUCGGAGUUCAUCA | Cleavage |
| osa-miR166b-5p | 4.5 | -1 | 1335 | 1354 | GGAAUGUUGUCUGGCUCGGGG  ::: :::::::: ::.:::::  CCCGGAGCCAGA-AAUAUUCC | Cleavage |
| osa-miR2103 | 4.5 | -1 | 931 | 952 | UUUCCCUCUCCGUGCGCGCUCG  :.:: ::::: ::::::.  GUCGUGCUCACGGCGAGGGAGU | Cleavage |
| osa-miR169q | 5 | -1 | 35 | 55 | UAGCCAAGGAGACUGCCCAUG  .:::::.::: ::::::.  AGUGGGCGGUCACCUUGGUGG | Translation |
| osa-miR5074 | 5 | -1 | 67 | 87 | GAAGGCCACCGUCGGGAUCGC  :::.:..:::::::: :  GGUCUCCUGGUGGUGGCCUAC | Cleavage |
| osa-miR5075 | 5 | -1 | 870 | 890 | UUCUCCGUCGCCGCCGUCCGC  : ::. :::::: :.:::: :  GAGGGAGGCGGCAAUGGAGCA | Cleavage |
| osa-miR528-5p | 5 | -1 | 736 | 756 | UGGAAGGGGCAUGCAGAGGAG  ::: :: :.::::.::: ::.  CUCGUCGGUAUGCUCCUACCG | Cleavage |
| osa-miR5519 | 5 | -1 | 121 | 141 | UGGCAGAAGUACUGGACUUAG  : ..:::: ::.: ::::::.  CCGGGUCCCGUGCCUCUGCCG | Cleavage |
| ***Os02g0221900*** | | | | | | |
| osa-miR2925 | 4.0 | -1.0 | 1486 | 1504 | UGGCGGCCGCGGGCUUCGU  ..:::::.::: ::::.:.  GUGAAGCUCGCCGCCGUCG | Cleavage |
| osa-miR5075 | 4 | -1 | 1383 | 1403 | UUCUCCGUCGCCGCCGUCCGC  :.::: ::::.:::::.:  CGCGGCGGGGGCGGCGGAGGA | Cleavage |
| osa-miR1430 | 4.5 | -1 | 446 | 466 | UGGUGAGCCUUCCUGGCUAAG  ::::.: .::::::.::.  ACCAGCCGGCGAGGCUCGCCG | Cleavage |
| osa-miR166j-5p | 4.5 | -1 | 197 | 217 | GAAUGACGUCCGGUCUGAAGA  :.:.::::::::: :::  AGAACGGGCCGGACGUCUUUC | Cleavage |
| osa-miR2927 | 4.5 | -1 | 657 | 679 | UGUCGUCGUCGAUGGAGCCCAUG  ::: :. ::::::::::.  AGACGGCGACGACGACGACGACG | Cleavage |
| osa-miR5075 | 4.5 | -1 | 640 | 660 | UUCUCCGUCGCCGCCGUCCGC  :: : ::::: :::::::::  GCCGCCGGCGACGACGGAGAC | Translation |
| osa-miR2926 | 5 | -1 | 661 | 680 | AGGUCGUCGACGUUGGUGCU  .::. :.::: ::::::: :  GGCGACGACGACGACGACGU | Translation |
| osa-miR5075 | 5 | -1 | 901 | 921 | UUCUCCGUCGCCGCCGUCCGC  :::: :::::::::.:  CGCGACGCCGGCGACGGGGCG | Cleavage |
| osa-miR5075 | 5 | -1 | 958 | 978 | UUCUCCGUCGCCGCCGUCCGC  : : .::::::::.:::. :  GAGAGCGGCGGCGGCGGGCAC | Cleavage |
| osa-miR5832 | 5 | -1 | 790 | 810 | UUGGCGGAGCGGUUGCUGUCA  :: :::..:.::::::::.  UGCAAGCGGCUGCUCCGCCGU | Cleavage |
| ***Os06g0565100*** | | | | | | |
| osa-miR1437b-3p | 2.5 | -1.0 | 323 | 346 | GUGCUGGCGAGCUCCGGUGCCGCA  :::::::::.:::.::.:  UCGCAAACCGGAGCUUGCCGGCGC | Cleavage |
| osa-miR1848 | 4.5 | -1.0 | 872 | 892 | CCUCGCCGGCGCGCGCGUGCA  ::. : :::::::::::. :  GGCGGGAGCGCGCCGGCGGCG | Cleavage |
| osa-miR2927 | 4.5 | -1 | 2 | 24 | UGUCGUCGUCGAUGGAGCCCAUG  :::::.: .:.::.::.:.  UGGAGGCUCUAGUGGCGGCGGCG | Cleavage |
| osa-miR3980a-3p | 4.5 | -1 | 1105 | 1125 | CUGGCCGAGGCCGUCGAUUCU  ..::::: ::.:::::: :  GAGGUCGACCGCUUCGGCCCG | Cleavage |
| osa-miR3980b-3p | 4.5 | -1 | 1105 | 1125 | CUGGCCGAGGCCGUCGAUUCU  ..::::: ::.:::::: :  GAGGUCGACCGCUUCGGCCCG | Cleavage |
| osa-miR2927 | 5 | -1 | 627 | 649 | UGUCGUCGUCGAUGGAGCCCAUG  : :::::. ::::: ::::.  ACCGCGCUCCGACGACGCCGACG | Cleavage |
| osa-miR3980a-3p | 5 | -1 | 559 | 579 | CUGGCCGAGGCCGUCGAUUCU  : ::::: : ::::::::  GCCACCGACGUCAUCGGCCAG | Translation |
| osa-miR3980b-3p | 5 | -1 | 559 | 579 | CUGGCCGAGGCCGUCGAUUCU  : ::::: : ::::::::  GCCACCGACGUCAUCGGCCAG | Translation |
| osa-miR5075 | 5 | -1 | 12 | 32 | UUCUCCGUCGCCGCCGUCCGC  :.::::::::.::: :.  AGUGGCGGCGGCGGCGGCGGC | Cleavage |
| osa-miR5075 | 5 | -1 | 1363 | 1383 | UUCUCCGUCGCCGCCGUCCGC  :::: :::::.:: .::.  UUCGACGCCGGCGGCGAGGAG | Cleavage |
| osa-miR827 | 5 | -1 | 82 | 102 | UUAGAUGACCAUCAGCAAACA  :: ::.:::::.::::  GUCGUGGUGGUGGUCGUCUAC | Cleavage |
