# Supplementary figures and images for: Diverse Roles of MAX1 Homologues in Rice
Source: Genes (Basel). 2020 Nov 13;11(11):1348. doi: 10.3390/genes11111348 (PMC7709044; doi:10.3390/genes11111348)

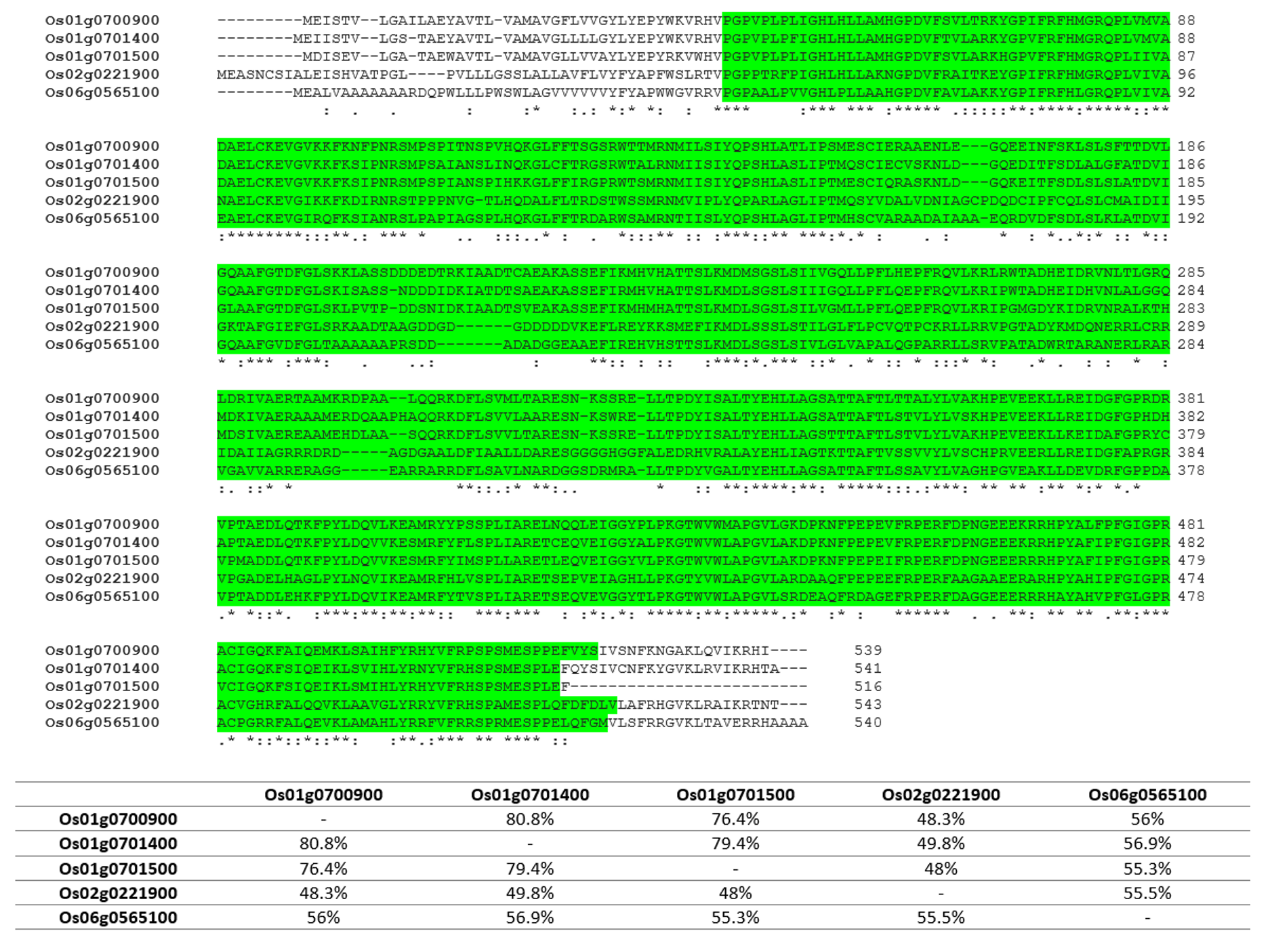

Supplement: Supplementary file 1 [file genes-11-01348-s001.zip › FigS1.tif]
